# Pellino 3 promotes the colitis‐associated colorectal cancer through suppression of IRF4‐mediated negative regulation of TLR4 signalling

**DOI:** 10.1002/1878-0261.13475

**Published:** 2023-06-27

**Authors:** Young‐Mi Kim, Hye‐Youn Kim, Huyen Trang Ha Thi, Jooyoung Kim, Young Jae Lee, Seong‐Jin Kim, Suntaek Hong

**Affiliations:** ^1^ Department of Biochemistry, Lee Gil Ya Cancer and Diabetes Institute Gachon University College of Medicine Incheon Korea; ^2^ GILO Institute GILO Foundation Seoul Korea; ^3^ Medpacto Inc. Seoul Korea

**Keywords:** colitis‐associated colorectal cancer, IRF4, Pellino 3, TLR4

## Abstract

The incidence of colitis‐associated colorectal cancer (CAC) has increased due to a high‐nutrient diet, increased environmental stimuli and inherited gene mutations. To adequately treat CAC, drugs should be developed by identifying novel therapeutic targets. E3 ubiquitin‐protein ligase pellino homolog 3 (pellino 3; Peli3) is a RING‐type E3 ubiquitin ligase involved in inflammatory signalling; however, its role in the development and progression of CAC has not been elucidated. In this study, we studied *Peli3*‐deficient mice in an azoxymethane/dextran sulphate sodium‐induced CAC model. We observed that Peli3 promotes colorectal carcinogenesis with increased tumour burden and oncogenic signalling pathways. Ablation of *Peli3* reduced inflammatory signalling activation at the early stage of carcinogenesis. Mechanistic studies indicate that Peli3 enhances toll‐like receptor 4 (TLR4)‐mediated inflammation through ubiquitination‐dependent degradation of interferon regulatory factor 4, a negative regulator of TLR4 in macrophages. Our study suggests an important molecular link between Peli3 and colonic inflammation‐mediated carcinogenesis. Furthermore, Peli3 can be a therapeutic target in the prevention and treatment of CAC.

## Graphical abstract

The authors found that a negative regulator of TLR4, IRF4, was degraded through K48‐mediated ubiquitination by Peli3 in response to LPS stimuli. As a result, the proinflammatory pathway was promoted by activating TLR4‐mediated signalling to induce colitis and CAC development. However, the loss or low levels of Peli3 allowed stabilisation of IRF4 protein to inhibit activation of NF‐κB, resulting in suppression of inflammatory signalling.

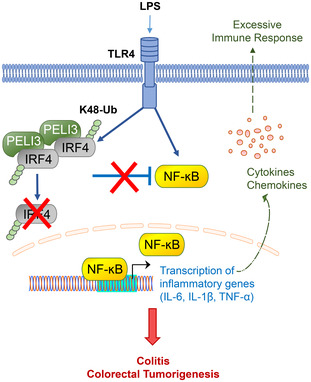

**Abbreviations**
ACF: aberrant crypt foci
AOM: azoxymethane
CAC: colitis‐associated colorectal cancer
DSS: dextran sulphate sodium
IBD: inflammatory bowel disease
IRF4: interferon regulatory factor 4
KO: knockout
LPS: lipopolysaccharide
MAPK: mitogen‐activated protein kinase
MPO: myeloperoxidase
NF‐κB: nuclear factor κB
PCR: polymerase chain reaction
Peli3: Pellino 3
PROTAC: proteolysis targeting chimera
STAT3: signal transducer and activator of transcription 3
TLR4: Toll‐like receptor 4
WT: wild type

## 1 Introduction

Colitis‐associated colorectal cancer (CAC) is the second most important cause of cancer‐associated death in developed countries [1]. According to previous studies, some cases of colorectal cancer are caused by inflammatory bowel disease (IBD), and patients with IBD, including ulcerative colitis and Crohn's diseases, are at high risk for developing CAC [2, 3]. Therefore, studying on the relationship between chronic inflammation and cancer development may extend our knowledge to the pathogenesis of CAC and, to some extent, sporadic colorectal cancer. In chronic inflammation in IBD, cytokines, chemokines, and matrix degrading enzymes are produced and initiate colon cancer development by inducing DNA damage, inhibiting apoptosis, promoting angiogenesis, or altering cell proliferation and cell survival, as well as inducing epigenetic alterations [4]. Infiltration of immune cells into tumours or pre‐neoplastic lesions also produces many kinds of cytokines that extend the localised inflammatory stimuli and enhances the survival and growth of premalignant cells through the activation of survival signalling pathways. However, the detail molecular mechanisms underlying the relationship between chronic inflammation and colon cancer development and progression need to be explored.

Inflammation is regulated by a variety of cell signalling pathways, including nuclear factor κB (NF‐κB), mitogen‐activated protein kinase (MAPK) and signal transducer and activator of transcription 3 (STAT3), that play critical roles in colorectal tumorigenesis [5, 6]. Activation of NF‐κB and MAPK signalling are primarily induced by toll‐like receptors (TLRs), which constitute a family of pattern‐recognition receptors that recognise pathogens or danger‐associated molecular patterns. All TLRs, except for TLR3, activate a canonical MyD88‐dependent pathway that recruits MyD88 to TLRs via TIR‐TIR domain interactions, forming scaffold proteins to recruit TNF receptor‐associated factor 6 (TRAF6) and TGFβ‐activated kinase 1 (TAK1), which form a complex with MyD88 [7, 8]. TAK1 activates MAPK and NF‐κB signalling, leading to the activation and nuclear translocation of transcription factors (activator protein‐1, NF‐κB) that stimulate transcription of inflammatory cytokine genes [7]. Therefore, dysregulation or activation of TLR expression are critical for IBD and CAC pathogenesis. In a mouse model, TLR4 knockout (KO) mice were protected against CAC, whereas TLR4 transgenic mice were prone to developing inflammatory neoplasia in the intestinal epithelium [9]. In humans, studies suggest that increased TLR4 signalling results in more aggressive CAC, whereas decreased TLR4 signalling is protective against CAC [10, 11]. Additionally, high TLR4 expression was correlated with the development of colonic neoplasia [12, 13]. Furthermore, silencing of TLR4 with RNA interference in xenograft models of CAC decreased metastasis to the liver [14].

Pellino (Peli) proteins, including Peli1, Peli2, and Peli3 (which splice variants 3a and 3b in human), are known as the E3 ubiquitin ligase in TLR signalling and interacts with interleukin‐1 receptor‐associated kinases (IRAKs), TRAF6 and TAK1 [15]. Therefore, Peli proteins are important regulators of TLR and the interleukin‐1 signalling pathway for stabilisation of signal complexes [16, 17, 18]. Although all Peli proteins are structurally similar, they exhibit different regulator effects on TLR and IL‐1R signalling by inducing K48‐linked polyubiquitination to degrade c‐Rel [19] or through K63‐linked polyubiquitination of TRAF‐6 [20]. Recent evidence has suggested that Peli3 is dispensable for TLR‐induced expression of proinflammatory cytokines, and that it negatively regulates TLR3 and virus‐mediated induction of type I interferon and related genes [21]. In lipopolysaccharide (LPS)‐mediated inflammatory signalling, Peli3 is an important regulator to prevent the excessive activation of cytokine expression by autophagy‐dependent degradation [22]. Peli3 has also been implicated as a critical regulator of atherosclerosis through its ability to suppress TLR4‐mediated induction of IFN‐β [23]. Another study also revealed that Peli3 in NOD2‐induced K63‐linked ubiquitination of RIP2 plays an important role in homeostatic control of intestinal inflammation [24]. Although recent reports have revealed the regulatory functions of Peli3 in TLR‐mediated regulation of intestinal inflammation, its relationship with colorectal carcinogenesis requires further study.

Interferon regulatory factors (IRFs) are member of transcription factors that interact a specific DNA sequence, namely the IFN‐stimulated response element, and play important functions in many immune processes [25]. One of its members, namely IRF4, is expressed in immune cells such as lymphocytes, dendritic cells and macrophages [26, 27]. Previous studies reported that the activation of macrophages with LPS induces translocation of IRF4 from the cytosol to the nucleus [28]. IRF4 and ETS‐like protein PU.1 have been shown to synergistically mediate transcriptional activation of the human IL‐1β gene in macrophages [29]. Moreover, IRF4 expression was induced by LPS and negatively regulated the production of proinflammatory cytokines in macrophages [30]. In contrast, IRF4 was also induced by MDP‐mediated NOD2, which attenuates the excessive activation of the TLR/NF‐κB signalling pathways [31, 32].

## 2 Materials and methods

### 2.1 Antibodies and reagents

Anti‐HA (sc‐805), Myc (sc‐40), Peli3 (sc‐376466), IRF4 (sc‐130921), PCNA (sc‐56), His (H‐3; sc‐8036), Ub (sc‐8017) and β‐catenin (H‐102; sc‐7199) antibodies were obtained from Santa Cruz (Santa Cruz, CA, USA). Antibodies against total IκBα (sc847), phospho‐IκBα (9246), Erk1/2 (9102), phospho‐Erk1/2 (9101), STAT3 (9132), and phosphor‐STAT3 (9145) were purchased from Cell Signaling (Danvers, MA, USA). Anti‐β‐actin (AC‐15, A1978), Flag (F3165) and LPS were obtained from Sigma‐Aldrich (St. Louis, MO, USA).

### 2.2 Generation of Peli3 KO mice

To generate the Peli3‐conditional KO mouse strain, a recombineering system was used to construct a conditional KO vector for targeting Peli3. A 10.6‐kb genomic DNA fragment possessing exon 1 of Peli3 was retrieved into the pLMJ235 plasmid. The loxP sequence and frt‐loxP‐Neo‐frt‐loxP cassette, having a positive selection marker (Neomycin‐resistant gene), were cloned 506‐bp upstream and 327‐bp downstream of exon 1, respectively. Then, linearised KO vector was transported into J1 ES cells (RRID:CVCL_6412; ATCC, Manassas, VA, USA) using electroporation. Targeted ES cells were injected into C57BL/6 blastocysts (Oriental Bio, Seongnam, Korea) and germline transmission of the targeted allele (Peli3^3f^) was confirmed by Southern blot analysis. To remove the Neo‐cassette in the Peli3^3f^ allele, Peli3^+/3f^ mice were crossed with Flp‐deleter mice (C57BL/6‐Tg(CAG‐Flpe)2Arte; Taconic Biosciences, Rensselaer, NY, USA). To generate Peli3 whole KO mice, Peli3^2f/2f^‐homozygotes were mated with transgenic mice expressing Cre recombinase under control of the β‐actin promoter (B6.FVB‐*Tmem163* ^*Tg(ACTB‐cre)2Mrt*^/EmsJ; Jackson Laboratory, Bar Harbor, ME, USA, Fig. S1A). Peli3 KO mice were genotyped by polymerase chain reaction (PCR) analysis of tail DNA with specific primers (Table S1). Peli3 KO mice or their wild type (WT) littermates were housed at a pathogen‐free facility maintaining 12 h/12 h circadian clock rhythm with standard chow. All animal experiments were performed in accordance with the guidelines approved by the Institutional Animal Care and Use Committees of Gachon University (LCDI‐2017‐0033, AAALAC‐accredited facility).

### 2.3 Induction of colitis and colorectal tumorigenesis

To induce CAC tumours, male mice (5–6 weeks of age) were administered with azoxymethane (AOM)/dextran sulphate sodium (DSS). Briefly, a single dose (8 mg·kg^−1^) of AOM (Sigma‐Aldrich) was intraperitoneally injected in Peli3 KO and WT control mice followed by three cycles of DSS (MW 40 kDa; MP Biomedicals, Irvine, CA, USA) dissolved in sterilised drinking water (cycle 1: 2%, 5 days; cycle 2: 1.5%, 5 days; cycle 3: 1.5%, 5 days). At 1, 4 or 18 weeks following DSS treatment, colon tissue was removed, opened longitudinally, and further processed for histological and immunohistochemical analysis.

### 2.4 Cell isolation and culture

For collection of elicited peritoneal exudate cells, mice were injected intraperitoneally with 3 mL of thioglycollate solution (4% wt/vol). After 4 days, cells were collected by flushing the peritoneum with 5 mL of ice‐cold phosphate‐buffered solution (PBS). Red blood cells were removed, and the remaining cells were washed using ice‐cold PBS. The collected peritoneal macrophages were cultured in DMEM (Welgene, Daegu, Korea) supplemented with 1% streptomycin/penicillin (Invitrogen, Carlsbad, CA, USA) and 10% heat inactivated foetal bovine serum at 37 °C in a CO_2_ incubator. Cell line was authenticated by short tandem repeat profiling within the last 3 years by ATCC and checked with MycoAlert PLUS Mycoplasma detection kit (Takara, Madison, WI, USA) before use.

### 2.5 Generation of stable cell lines using lentiviral system

To generate lentivirus, Lenti‐HEK293T packaging cells (RRID:CVCL_0063) were transfected with a pCAG lentiviral vector (GFP, HA‐Peli3) using Lipofectamine 2000 reagent (Invitrogen) as described previously [33]. The transfected cells were maintained in DMEM that contained 10% foetal bovine serum, and produced lentiviruses were collected after 48 h using 0.45‐μm filters. Then, peritoneal macrophages were infected with different lentiviral supernatants thrice every 12 h with polybrene (8 μg·mL^−1^) (Sigma‐Aldrich). Peli3 expression was measured by Western blotting with anti‐HA antibody. For generation of knockdown cell line for IRF4, shRNA was inserted into a lentiviral shRNA vector. The shRNA sequences for IRF4 are listed in Table S2.

### 2.6 Validation of gene expression using quantitative real‐time PCR

Total RNA was isolated with TRIzol reagent, and cDNA was generated with random hexamers using Super Script II (Invitrogen). The quantification of cytokine transcripts was performed with SYBR‐green Premix Ex‐Tag II (Takara) using real‐time quantitative PCR on Applied Biosystem Prism 7900HT sequence detection system (Thermo Scientific, Rockford, IL). The primer sequences for PCR are listed in Table S3. The relative expression level was analysed using the comparative ddCt method with cyclophilin as control [34]. The experiments were performed in triplicates and expressed as the mean ± standard deviation (SD).

### 2.7 Bacterial DNA extraction from caecal sample and microbiota analysis

After mice were killed, the contents of caecum were immediately placed in liquid nitrogen and stored at −80 °C until analysis. Bacterial DNA was collected using DNA stool isolation kit (Qiagen, Valencia, CA, USA) according to the manufacturer's instructions [35]. The 16S rRNA of each group was analysed with species‐specific qRT–PCR primers. The relative abundance of bacterial groups was expressed as a ratio of eubacteria. Bacterial primers used for qRT‐PCR are listed in Table S3.

### 2.8 Immunoblotting and immunoprecipitation

Total proteins were isolated with a lysis buffer (25 mm HEPES [pH 7.5] 150 mm NaCl, 1% Triton X‐100, 10% glycerol, 5 mm EDTA and a protease inhibitor cocktail) for 30 min and soluble fractions were collected by removing cell debris with centrifugation. The extracted protein concentrations were calculated by the BCA method (Pierce, Rockford, IL, USA). Then, protein lysates were separated by SDS/PAGE followed by transfer onto the polyvinylidene difluoride membrane. The membranes were incubated overnight with primary antibodies and assessed by chemiluminescence method with secondary antibody according to the manufacturer's protocol (Pierce). For protein precipitation, protein lysates were mixed with specific antibodies at 4 °C overnight and incubated with protein A/G beads (Bioprogen, Daejeon, Korea) for 3 h rotation. The precipitated beads were washed trice with a washing buffer (25 mm Tris–HCl, pH 8.0, 150 mm NaCl, 1% Triton X‐100), and associated proteins were extracted with 2× Tris‐Glycine SDS buffer at 100 °C for 5 min. Then, eluted proteins were analysed with Western blotting with specific antibodies.

### 2.9 Histological analysis and immunohistochemistry

Formalin‐fixed paraffin‐embedded tissues were deparaffinised and hydrated by standard protocol for haematoxylin–eosin (H&E) and special staining [36]. For immunohistochemistry, slide‐mounted tissue sections were microwaved for 20 min with 10 mm citrate buffer (pH 6.0) containing 0.01% Tween 20 and incubated with 0.3% hydrogen peroxide for 10 min. The slides were then incubated overnight with anti‐PCNA, CD163, Ly6G, CD3, CD11c, B220 and myeloperoxidase (MPO) antibodies diluted in 1% bovine serum albumin at 4 °C. After 24 h, the slides were incubated for 30 min with the secondary antibody. The stained proteins in the sections were visualised with diaminobenzidine (Dako, Carpinteria, CA, USA), counterstained with Hematoxylin QS (H‐3404; Vector Laboratories, Burlingame, CA, USA), and processed by a mounting medium. Analysis was performed with a confocal microscope at the Core‐facility for Cell to *In‐vivo* imaging of Gachon University. Quantitative analysis of the staining‐positive cells was calculated with metamorph software (Universal Imaging Corp, Burnaby, BC, Canada).

### 2.10 Ubiquitination assay

To confirm Peli3‐dependent ubiquitination of IRF4, IRF4 DNA was co‐transfected into HEK293 cells with WT or mutant ubiquitin and Peli3 constructs and treated with 10‐μm proteasome inhibitor MG132 (Calbiochem, Bedford, MA, USA) for 6 h. The proteins were extracted using NP‐40 lysis buffer and incubated with specific primary antibodies at 4 °C for 12 h. For immunoprecipitation of ubiquitinated IRF4 proteins, mixtures were further incubated with protein A/G‐agarose beads at 4 °C for 1 h. Then, the precipitated beads were washed with a lysis buffer thrice and analysed using Western blotting with specific antibodies.

### 2.11 Statistical analysis

Differences between experiment groups were compared using Student's *t*‐test (two‐tailed), and error bars indicate the SD of the mean value. One‐way analysis of variance and Bonferroni's correction were performed to compare data between three or more groups. Data are presented as mean ± SD unless otherwise indicated. The *P* values of < 0.05 were used to judge the statistical significance of experiment.

## 3 Results

### 3.1 Ablation of Peli3 reduces the development of CAC

**Fig. 1:**
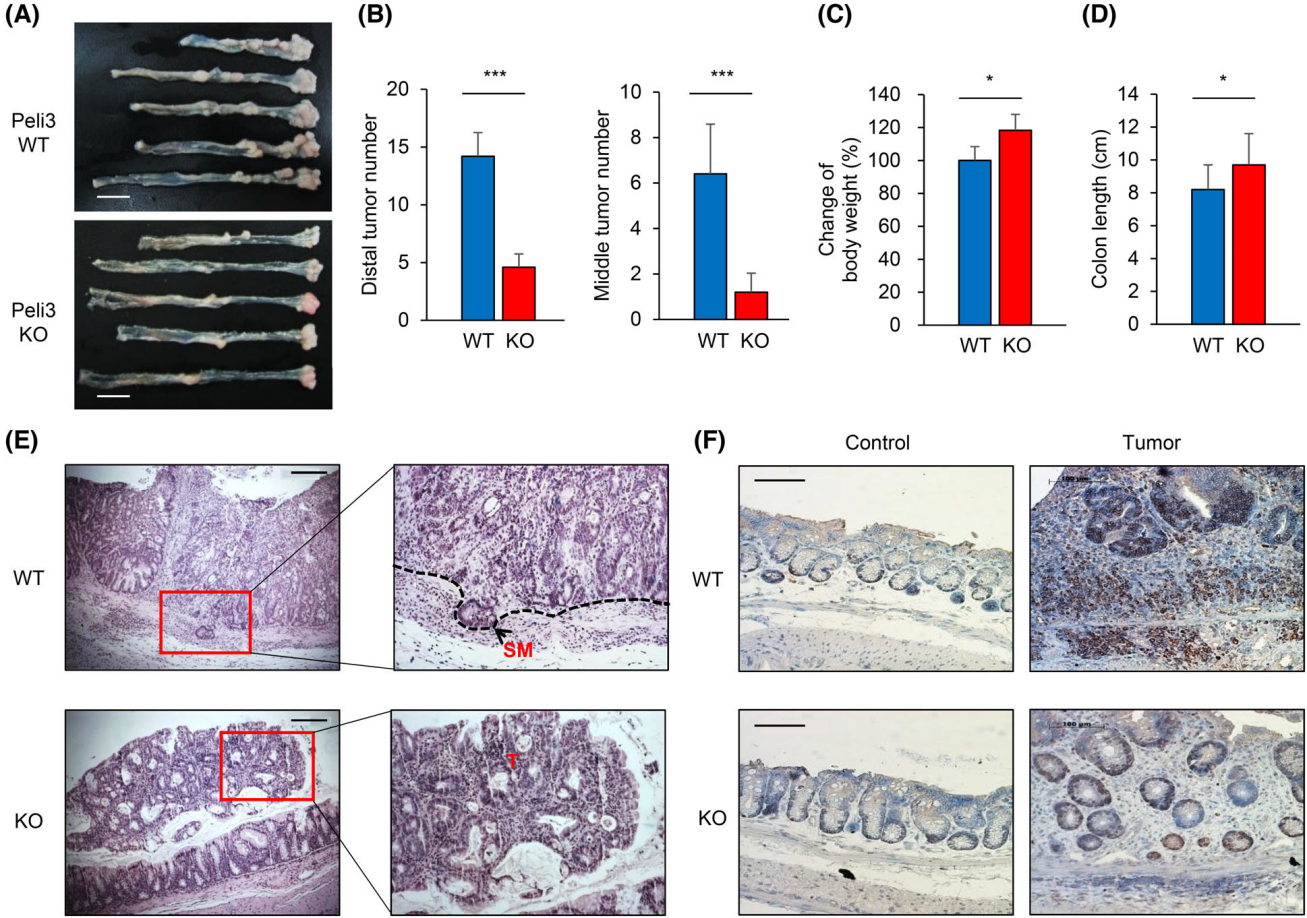
Ablation of Peli3 suppresses the development of AOM/DSS‐induced CAC. (A) After 18 weeks of AOM/DSS administration, colonic tissues were harvested, and their lengths and tumour appearance were compared (*n* = 6 per group). Representative images of the colon from the caecum to the proximal rectum of Peli3 WT or KO mice. Scale bar = 10 mm. (B) The number of tumours was counted from the distal or middle region of colonic tissues. (C, D) Body weight and colon length of Peli3 WT or KO mice were measured. (E) Representative images for haematoxylin and eosin (H&E)‐stained colonic tissues. SM, sub‐mucosa; T, tumour. Scale bar = 200 μm. (F) The proliferating cells were checked with PCNA staining of normal or tumour areas in each mouse. Scale bar = 100 μm. All experiments were repeated at least three times. All *P* values were calculated using unpaired two‐tailed Student's *t*‐tests. Results are presented as mean ± standard deviation from three independent experiments. *P < 0.05; ***P < 0.001.

To investigate the novel biological roles of Peli3 in colitis‐associated tumour development, we generated whole Peli3 KO mice by crossing with a β‐actin Cre driver mouse (Fig. S1A). The deletion of the Peli3 gene was confirmed by genotyping, and the loss of Peli3 in colonic tissue was confirmed by Western blotting (Fig. S1B,C). Then, Peli3 WT and KO mice were applied to the chemical‐induced colon carcinogenesis protocol. Briefly, both strains were administered with a single intraperitoneal injection of AOM followed by three cycles of DSS treatment and maintained with regular water during the experiment. Mice were killed, and colorectal tumours were collected after 18 weeks (Fig. S1D). In three independent experiments, the incidence of colonic tumours was observed in both WT and Peli3 KO mice and were most frequently observed in the middle and distal colon (Fig. 1A,B). Interestingly, ablation of Peli3 led to significantly less number of tumour incidence than in WT mice. Weight loss and shortening of the colon, which are characteristic of colon carcinogenesis, improved in Peli3 KO mice (Fig. 1C,D). Histologically, these tumours were diagnosed as well differentiated adenocarcinoma in WT mice with sub‐mucosal invasion, whereas tumours from Peli3 KO mice were small tubular non‐invasive adenomas (Fig. 1E). Moreover, the number of proliferating cells was significantly reduced in Peli3 KO tumour tissues compared with those in WT mice (Fig. 1F, Fig. S1E). Consistent with Peli3‐dependent CAC induction, expression of Peli3 was gradually increased during CAC development in mouse model (Fig. S1F). These results suggest the critical role of Peli3 in promoting chemical‐induced colorectal tumorigenesis.

### 3.2 Peli3‐deficient mice are resistant to DSS‐induced colitis

Aberrant crypt foci (ACF) are recognised as a hallmark of early malignant lesions in colorectal cancer [37, 38]. To evaluate the role of Peli3 in the development of CAC, we assessed the development of ACF in Peli3 WT and KO mice (Fig. S2A). Four weeks after DSS treatment, WT mice had significant loss of body weight and a shorter colon compared to KO mice (Fig. S2B,C). Numerous nodular, polypoid and caterpillar‐like tumours developed earlier, and the number of ACF per mouse was significantly increased in WT mice compared with KO mice (Fig. S2D,E). Histopathological examination of colon sections showed more severe epithelial erosions and obvious sub‐mucosal oedema in WT mice (Fig. S2F). Consistent with hyperplasia in WT mice colon, immunostaining of colon sections with PCNA antibody showed increased numbers of proliferating epithelial cells in WT mice compared with KO mice (Fig. S2G). Consistent with the histological differences, β‐catenin signalling was reduced in the colon of KO mice (Fig. S2H). These results revealed that Peli3 increased susceptibility to CAC development induced by AOM/DSS administration.

**Fig. 2:**
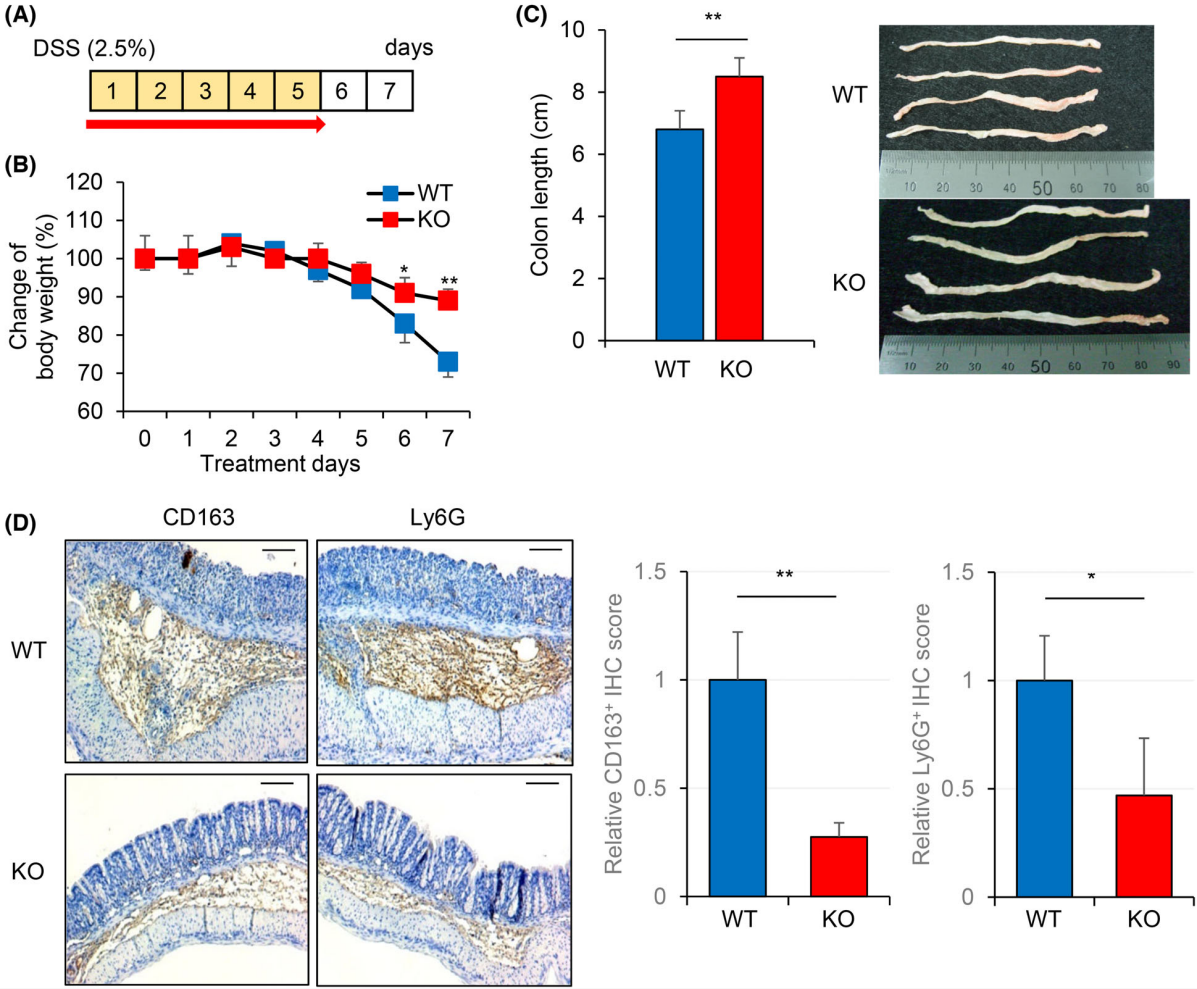
Peli3 promotes acute colitis‐induced by DSS. (A) Experimental protocol for acute colitis model. Mice were administered with 2.5% DSS for 5 days (*n* = 6 per group). Then, mice were killed after 2 days, and colonic tissues were collected for further analysis. (B) Body weights were measured every day for 1 week. (C) After collection, the colon length of Peli3 WT and KO mice were measured. Representative pictures of the colon are shown. Unit of scale bar is mm. (D) Representative immunohistochemical images of immune cells are shown. Slides were stained with anti‐CD163 for macrophages or anti‐Ly6G for neutrophils. Scale bar, 200 μm. Results are presented as mean ± standard deviation from three independent experiments. Significance between groups was analysed with Student's *t*‐test. *P < 0.05; **P < 0.01.

Because Peli3 is involved in the development of ACF at the early stages of colon cancer, we assessed the function of Peli3 in colitis‐induced inflammation. For this purpose, age‐matched Peli3 WT and KO mice were chosen to create a DSS‐induced acute colitis model. Mice from two strains were fed with 2.5% DSS in drinking water for 5 days, and pathological change was determined by measuring body weight and colon length, which are regarded as macroscopic measurements of tissue remodelling in short‐term inflammation (Fig. 2A). On day 7 after DSS treatment, there was a significant difference in colon length and weight loss between Peli3 WT and KO mice (Fig. 2B,C). Consistent with this result, IHC analysis of colon sections showed more hyperplasia and infiltration of various inflammatory cells, including macrophage (CD163), neutrophil (Ly6G), dendritic cell (CD11c), T cell (CD3) and B cell (B220), in WT mice compared with KO mice (Fig. 2D, Fig. S3A). When comparing the degree of infiltration of immune cells, macrophage showed most dramatic change in damaged condition. Consistent with reduced neutrophil recruitment, the degree of MPO‐positive sections was slightly decreased in Peli3‐deficient samples (Fig. S2B). These data revealed the role of Peli3 in promoting pathological inflammatory response of colonic tissue by mainly modulating macrophage activity.

### 3.3 Peli3 activates inflammatory pathways during CAC development

**Fig. 3:**
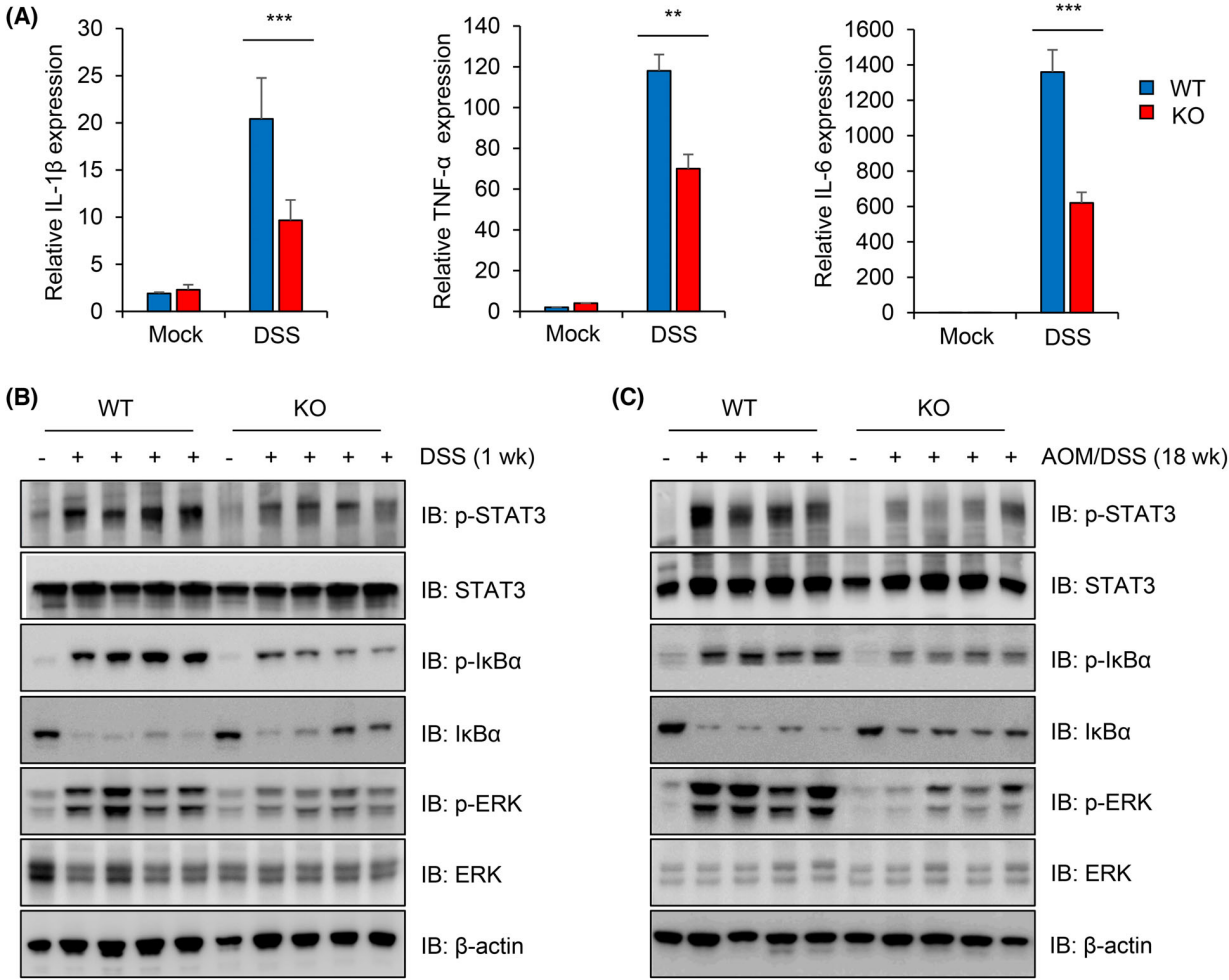
Peli3 activates the inflammatory pathway during the CAC development. (A) Expression levels of inflammatory cytokine genes were detected using qRT‐PCR in DSS‐treated colonic tissues of Peli3 WT or KO mice (*n* = 3 per group). Levels of cyclophilin were used as normalisation control. (B, C) To determine the activation of inflammatory signalling, colonic tissues were harvested after 1 or 18 weeks of AOM/DSS treatment. Levels of protein were detected using Western blotting with specific antibodies. All experiments were repeated at least three times. Results are presented as mean ± standard deviation from three independent experiments. Significance between groups was analysed with Student's *t*‐test. **P < 0.01; ***P < 0.001.

To further confirm the weakened inflammatory signalling in Peli3 KO mice, transcript levels of inflammatory mediators were analysed in both DSS‐treated colonic tissue and colonic tumours using real‐time PCR. The expression of proinflammatory genes, such as IL‐6, TNF‐α and IL‐1β was significantly lower in the colon of KO mice than in WT mice in both acute colitis and tumour samples (Fig. 3A, Fig. S4A). IL‐6 is an aetiological factor of CAC development through the promotion of the activation of the STAT3 signalling pathway [39, 40]. Increased expression of IL‐1β and TNF‐α was also implicated in colorectal cancer development and progression [41]. Therefore, reduced expression of these proinflammatory cytokines in Peli3 KO mice contributed to the decreased risk of developing CAC.

The expression of tumorigenic and inflammatory genes is driven by signal transduction pathways such as NF‐κB, MAPK and STAT3 [40, 42]. To investigate whether these pathways were deregulated in KO mice, we analysed inflammatory signalling activation in AOM/DSS‐treated colonic tissues of Peli3 WT and KO mice by western blotting. Consistent with weakened inflammation in Peli3 KO mice, the levels of phospho‐ERK, IκBα, and STAT3 in Peli3 KO mice were significantly downregulated as compared to those in WT mice (Fig. 3B). Activation of NF‐κB, STAT3 and ERK signalling pathways were also highly induced in tumour tissues of Peli3 WT mouse compared to those in KO mouse (Fig. 3C). These data indicate a strong association between Peli3‐mediated activation of inflammatory signalling pathways and colorectal carcinogenesis.

The proinflammatory condition of gut induced the disruption of microbiota homeostasis which is associated with reduction in probiotic bacteria and increase in pathogenic ones [43, 44]. To confirm the effect of Peli3 on microbiome homeostasis, we checked the change in distribution of microbiome after DSS treatment. As shown in Fig. S4B, pathogenic *Escherichia coli* subgroups and Delta/Gamma proteobacteria were markedly increased in DSS‐treated WT mice. However, imbalance of pathogenic microorganisms was disappeared in Peli3 KO mice. In contrast, probiotic bacteria, such as *Lactobacillus* and *Firmicutes* were decreased in WT mice and restored in KO mice. These results suggested that Peli3 is a critical mediator in DSS‐induced colitis by disrupting intestinal microbiota homeostasis.

### 3.4 Depletion of Peli3 suppresses the inflammatory response in macrophages

**Fig. 4:**
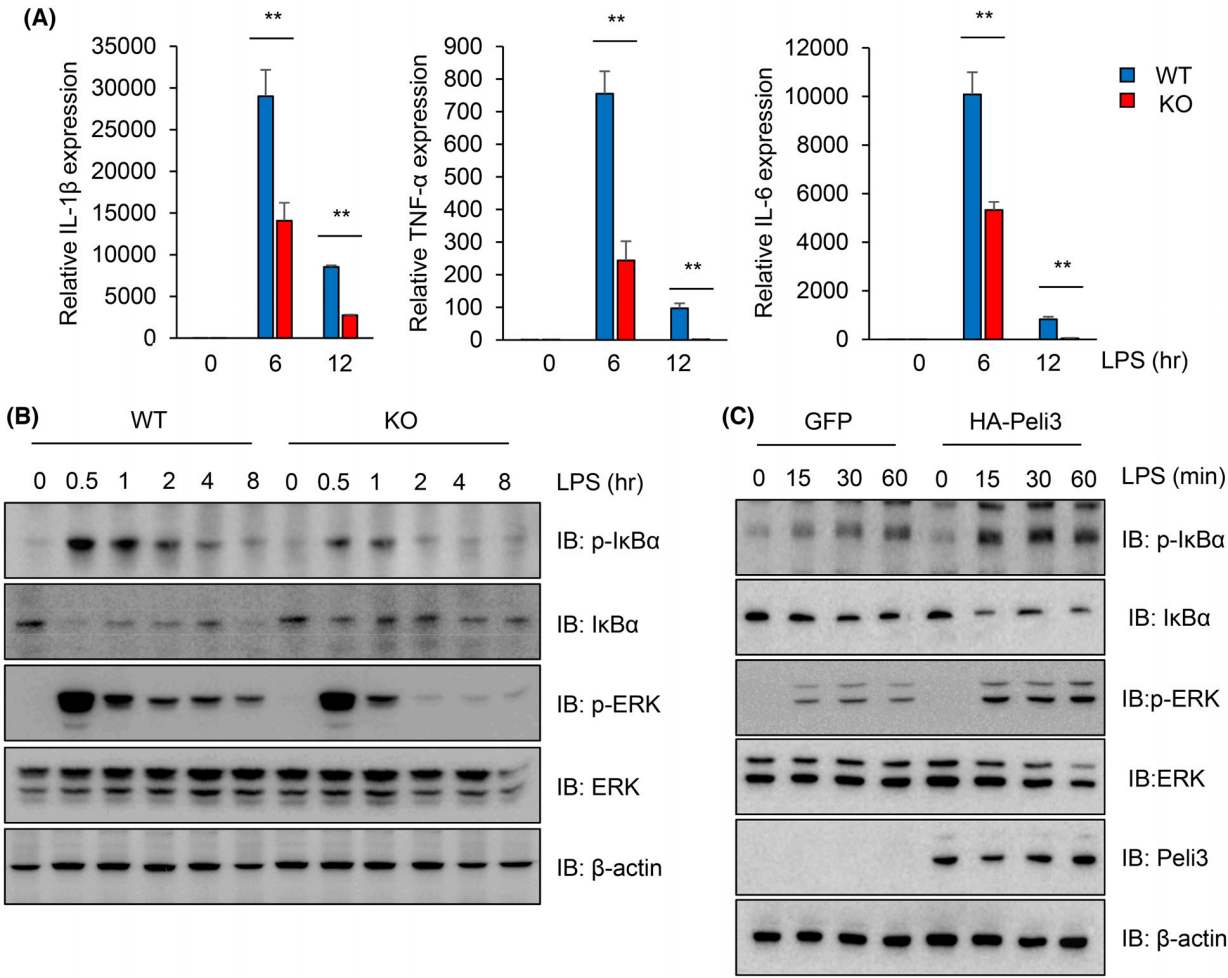
Depletion of Peli3 suppresses the inflammatory response in macrophages. (A) To evaluate the role of Peli3 in macrophages, we isolated peritoneal macrophages from Peli3 WT or KO mice (*n* = 3 per group). Then, we treated macrophages with LPS and isolated the total RNA. The expression level of inflammatory cytokine genes was detected using qRT‐PCR using macrophages of WT or KO mice. Levels of cyclophilin gene were used as normalisation control. (B) To determine the activation of inflammatory signalling, peritoneal macrophages were harvested after treatment with LPS. The protein extracts were prepared with lysis buffer, and levels of each protein were detected using Western blotting with specific antibodies. (C) To confirm the direct effect of Peli3 on inflammatory signalling activation, the Peli3 gene was introduced in peritoneal macrophages. Cells were treated with LPS, and protein was extracted with a lysis buffer. Each protein was detected using Western blotting with specific antibodies. All experiments were repeated at least three times. Results are presented as mean ± standard deviation from three independent experiments. Significance between groups was analysed with Student's *t*‐test. **P < 0.01.

Toll‐like receptor signalling plays a critical role in intestinal homeostasis. Injured epithelial cells result in exposure of several TLR ligands produced by commensal microbes caused by the disruption of the mucosa [45]. TLR4‐ and MyD88‐deficient mice developed severe colonic injury due to uncontrolled intestine homeostasis induced by DSS administration [9, 46]. Because TLR4‐mediated NF‐κB and MAPK signalling are classical inflammatory pathways that modulate the expression of proinflammatory cytokines, we evaluated the effects of Peli3 on LPS‐stimulated mouse peritoneal macrophages. As shown in Fig. 4A, loss of Peli3 significantly suppressed proinflammatory cytokine expression induced by LPS in peritoneal macrophages. Similarly, treatment with LPS rapidly increased phosphorylation of IκBα and ERK after 30 min and gradually decreased after 2 h; however, Peli3 KO peritoneal macrophages significantly reduced phosphorylation of IκBα and ERK (Fig. 4B). To further confirm the effects of Peli3 on the activation of LPS‐induced NF‐κB and MAPK signalling, Peli3 overexpressing peritoneal macrophage or control cells were treated with LPS. As shown in Fig. 4C, overexpression of Peli3 increased phosphorylation of IκBα and ERK upon LPS stimulation compared with control cells. These results confirm that Peli3 positively regulates LPS‐induced NF‐κB and MAPK signalling pathways in macrophages.

### 3.5 Degradation of IRF4 is associated with Peli3‐mediated activation of inflammatory pathways

IRF4 is one of the regulatory molecules that attenuate excessive activation of TLR mediated NF‐κB signalling [30, 31]. IRF4 inhibits TLR‐induced activation of NF‐κB via interaction with MyD88 and TRAF6 upon stimulation of MDP and LPS. Therefore, we hypothesised that Peli3 may regulate LPS‐induced inflammatory signalling through inhibition of IRF4. To investigate the effects of Peli3 on IRF4 expression, we measured the mRNA level of IRF4 after treatment with various TLR ligands in mouse peritoneal macrophages. TLR ligand treatment did not result in a significant difference in the levels of IRF4 mRNA in peritoneal macrophages in Peli3 KO and WT mice (Fig. S5). This result indicates that Peli3 does not regulate IRF4 at the transcriptional level.

**Fig. 5:**
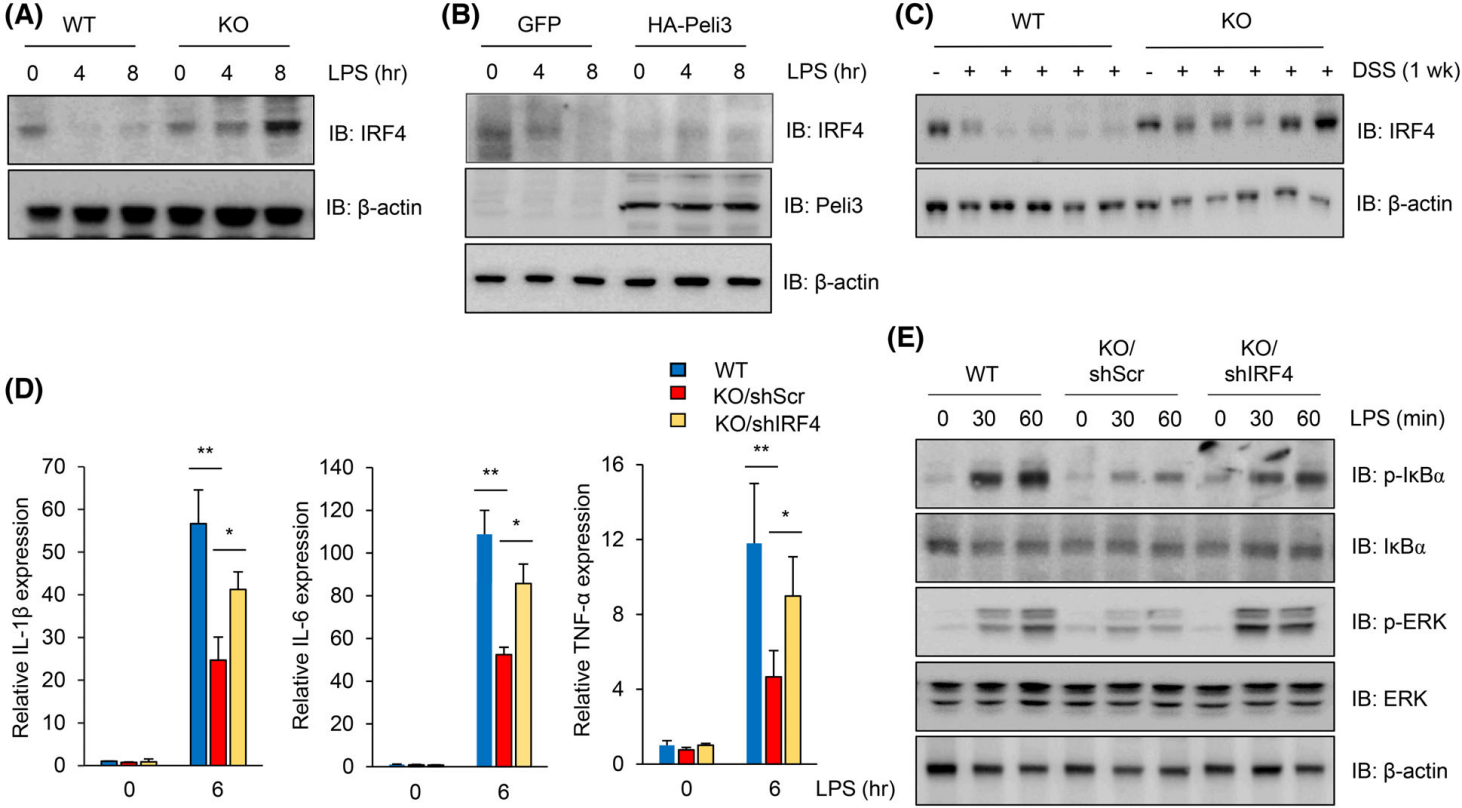
Peli3 activates inflammatory signalling through degradation of IRF4 protein. (A) To measure the effects of Peli3 on the levels of IRF4 protein, peritoneal macrophages were treated with LPS. Then, whole protein was extracted, and the level of IRF4 was measured with IRF4 antibody. (B) Control or Peli3‐stably expressing peritoneal macrophages were treated with LPS, and protein was extracted with a lysis buffer. IRF4 and Peli3 were detected using Western blotting with specific antibodies. The band intensity of each protein was normalised with that of β‐actin. (C) To measure the level of IRF4 in colonic tissues, proteins were extracted after treatment with DSS for 1 week. Then, whole protein was extracted after grinding the colonic tissues, and the level of IRF4 was detected with the specific antibody. (D) To confirm the negative regulation of IRF4 in inflammatory signalling, IRF4 was depleted in Peli3 KO peritoneal macrophages using lentiviral shRNA. Each cell was treated with LPS for 6 h, and total RNA was extracted. Expression of inflammatory cytokines was measured with qRT‐PCR with specific primers. Levels of cyclophilin gene were used as normalisation control. (E) Protein extracts were prepared from Peli3 KO or IRF4‐depleted macrophages after treatment with LPS. Proteins were separated with SDS/PAGE and detected with specific antibodies. All experiments were repeated at least three times. Results are presented as mean ± standard deviation from three independent experiments. Significance between groups was analysed with Student's *t*‐test. *P < 0.05; **P < 0.01.

Next, we determined the expression of IRF4 upon stimulation by LPS in both Peli3 KO and WT peritoneal macrophages. Interestingly, after treatment with LPS, IRF4 protein in WT macrophages disappeared (Fig. 5A), whereas IRF4 protein was retained in Peli3 KO peritoneal macrophages. Conversely, ectopic expression of Peli3 in macrophage cells markedly suppressed IRF4 expression after treatment with LPS (Fig. 5B). Additionally, colitis‐induced degradation of IRF4 protein in WT mice colonic tissues was not evident in KO mice colonic tissues in acute colitis and early stages of CAC (Fig. 5C, Fig. S6). To validate the IRF4‐mediated inhibition of inflammatory signalling in Peli3 KO mice, we generated a knockdown cell line for IRF4 in Peli3 KO mice macrophages (Fig. S7). As shown in Fig. 5D, depletion of IRF4 in Peli3 KO mice peritoneal macrophages restored the expression of inflammatory genes. Consistent with this result, ablation of IRF4 in Peli3 KO mice macrophages recovered NF‐kB and ERK signalling (Fig. 5E). These results suggest that Peli3 promotes inflammatory signalling through degradation of IRF4 at the post‐translational level.

### 3.6 Peli3 mediates K48‐linked polyubiquitination and degradation of IRF4

**Fig. 6:**
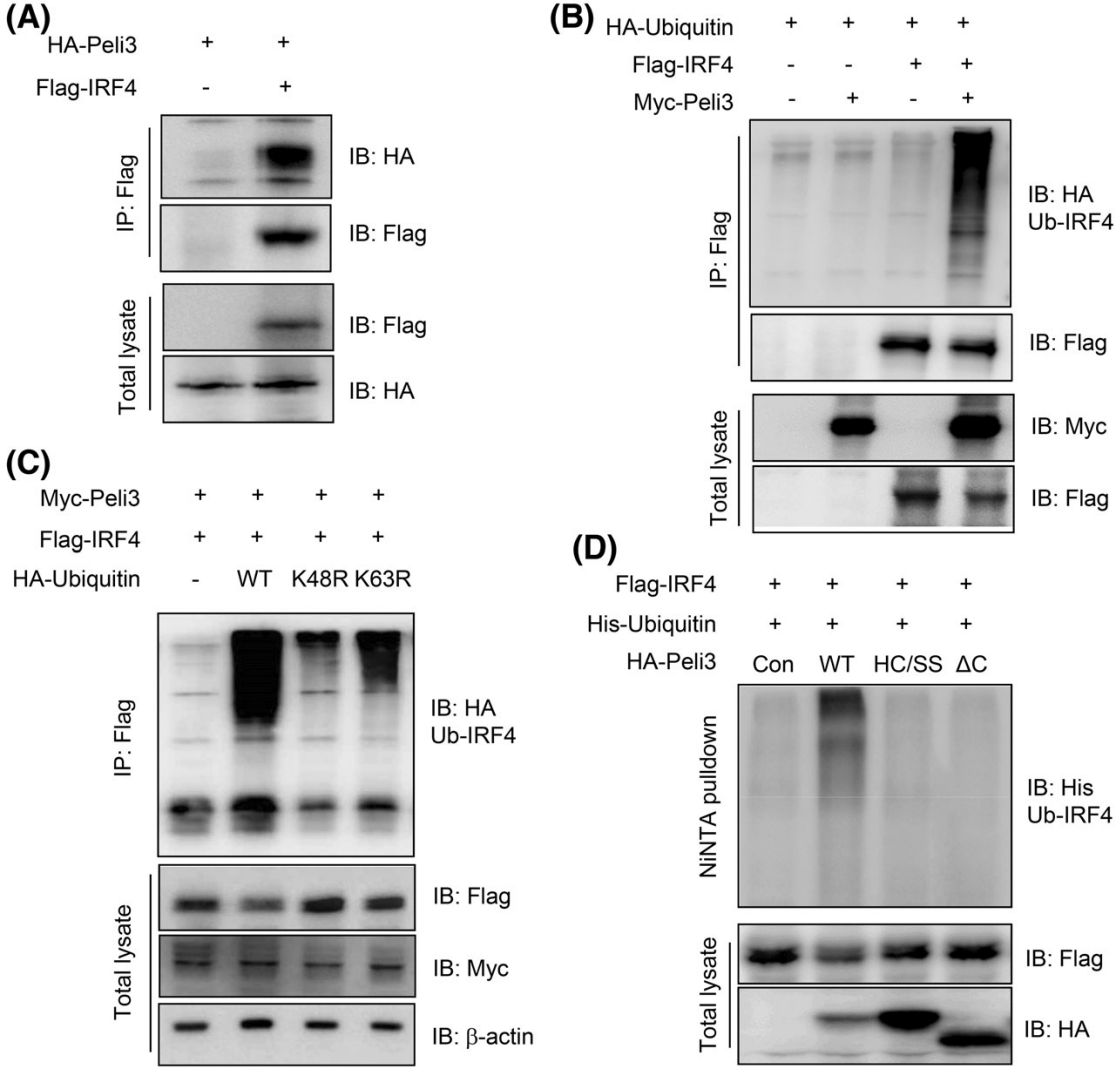
Peli3 induces the degradation of IRF4 through K48‐mediated ubiquitination. (A) To detect the direct interaction of Peli3 and IRF4, Peli3 and IRF4 DNA constructs were transfected into HEK293 cells. After 48 h, cell lysates were prepared, and protein complexes were precipitated with an anti‐Flag antibody. The immunoprecipitated Peli3 was measured with the anti‐HA antibody, and expression of transfected proteins was detected with total lysate. (B) To determine Peli3‐mediated ubiquitination of IRF4, Peli3 and IRF4 constructs were transfected with HA‐ubiquitin constructs into HEK293 cells. After 48 h, cells were lysed and applied to immunoprecipitation. Ubiquitinated IRF4 was precipitated with Flag antibody and detected with HA antibody. Expression of transfected protein was confirmed with the specific tagging antibody. (C) To determine the type of ubiquitination, IRF4 constructs were transfected with WT, K48R or K63R mutant ubiquitin constructs. After 48 h, protein lysates were immunoprecipitated with anti‐Flag antibody, and ubiquitinated IRF4 was detected with anti‐HA antibody. (D) To evaluate the role E3 ubiquitin ligase activity of Peli3 in ubiquitination of IRF4, WT or mutant Peli3 constructs were transfected with ubiquitin construct. Then, protein lysates were immunoprecipitated with NiNTA, and ubiquitinated IRF4 was detected with anti‐His antibody. All experiments were repeated at least three times.

To investigate whether Peli3 affects the stability of IRF4 at the post‐translational level as E3 ubiquitin ligase, we first determined the interaction between Peli3 and IRF4 by performing an immunoprecipitation assay. As shown in Fig. 6A, IRF4 was strongly immunoprecipitated with Peli3. To examine whether IRF4 degradation was induced by polyubiquitination, we reconstituted the ubiquitination assay for IRF4. Cell lysates were extracted with lysis buffer containing 2% SDS to exclude nonspecific binding to ubiquitin, and transferred membranes were treated with denaturation buffer containing 6 m of guanidine chloride [47]. Peli3 significantly induced ubiquitination and degradation of IRF4 (Fig. 6B). To verify the type of ubiquitination of IRF4 by Peli3, we co‐transfected K48R or K63R ubiquitin mutant constructs in combination with Peli3 and IRF4 (Fig. 6C). Peli3 strongly induced polyubiquitination of IRF4 in the presence of the WT‐ubiquitin construct. However, this modification completely disappeared with the mutant K48R ubiquitin and was slightly affected by the mutant K63R ubiquitin construct. Because the RING‐like domain in the Peli protein is necessary for E3 ubiquitin ligase activity, we further examined whether the E3 ligase activity of Peli3 is important for ubiquitination and degradation of IRF4. We co‐transfected Peli3 WT mice with a RING‐like domain‐deletion (HA‐Peli3ΔC) or a HA‐Peli3(HC/SS) mutant construct, which are important for E3 ubiquitin ligase activity within the RING‐like domain. As shown in Fig. 6D, both Peli3‐ΔC and Peli3‐HC/SS mutants inhibited ubiquitination of IRF4 and restored IRF4 expression. These results suggest that Peli3 negatively regulates the stability of IRF4 through K48‐mediated polyubiquitination and positively induces inflammatory signalling and CAC.

## 4 Discussion

**Fig. 7:**
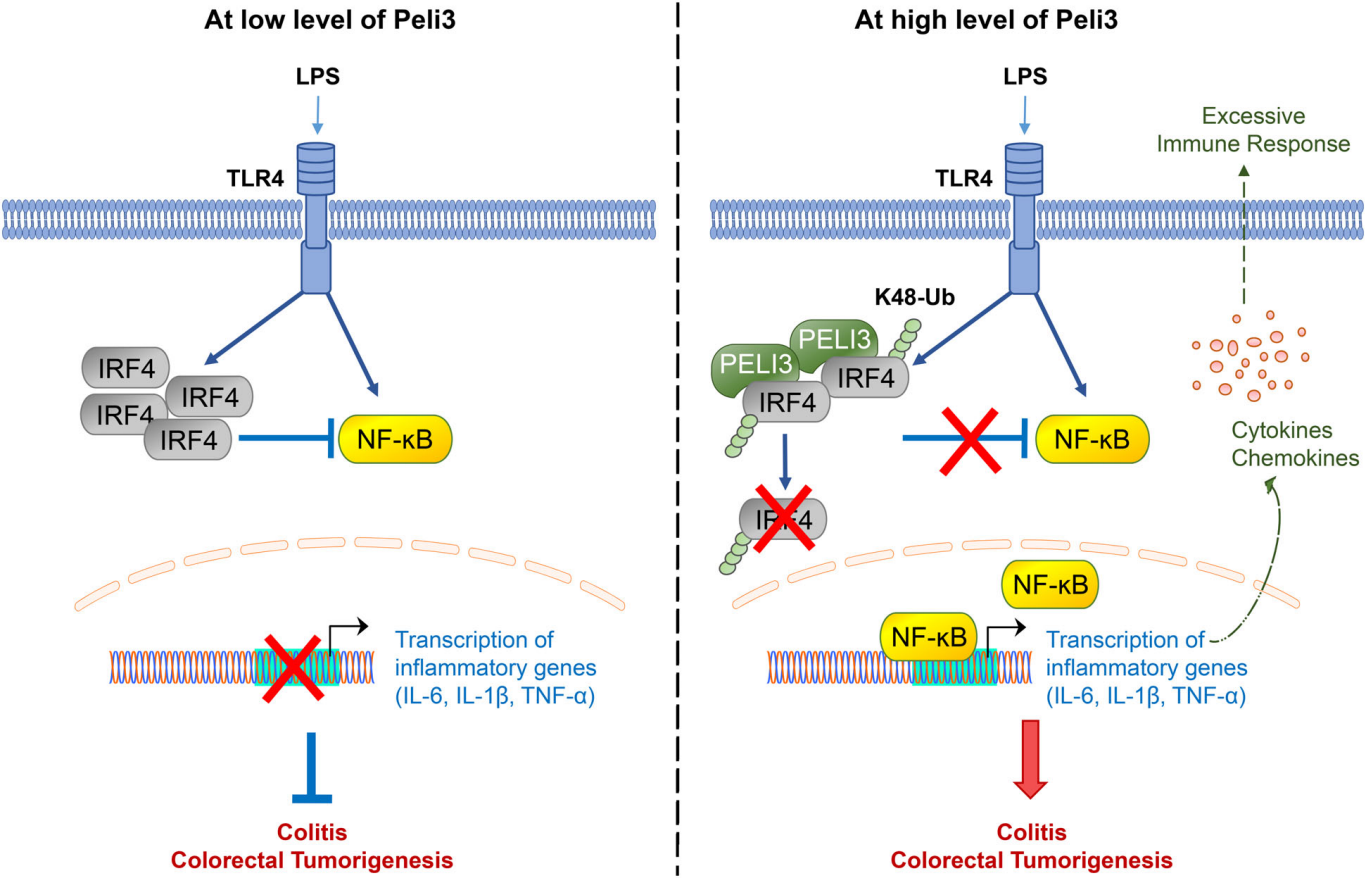
A proposed model for the role of Peli3 in CAC development. With a high level of Peli3, IRF4 was degraded through K48‐mediated ubiquitination by Peli3 up on response to LPS stimuli. As a result, the proinflammatory pathway was promoted by activating TLR4‐mediated signalling to induce colitis and CAC development. However, the loss or low levels of Peli3 allowed stabilisation of the IRF4 protein to inhibit activation of NF‐κB, resulting in suppression of inflammatory signalling.

Herein, we identified the oncogenic role of Peli3 in inflammation‐associated colon tumorigenesis using an AOM/DSS‐induced CAC model. Depletion of Peli3 suppressed the activation of proinflammatory signalling due to high levels of IRF4, leading to reduced CAC carcinogenesis (Fig. 7). Peli3 promotes the development of CAC through ubiquitin‐dependent degradation of IRF4 to enhance the expression of inflammatory cytokines. Because tight regulation of inflammatory signalling is critical for homeostasis of immune system, several negative regulators including A20, RP105 and small heterodimer partner are involved in suppression of excessive TLR activation [48, 49, 50]. These regulators modulate the TLR signalling by preventing interaction of ligand and receptor, recruitment of adaptor proteins and inhibition of post‐translational modification. Due to association of aberrant expression of negative regulators and many human diseases, it will be interesting approach to analyse the Peli3‐mediated suppression of these regulators in CAC development. By modulating these negative signals in inflammation, Peli3 may functions as an oncogene of colon cancer, especially working at the cancer microenvironment. Other RING‐type E3 ubiquitin ligases are also involved in the development of colorectal cancer [51, 52, 53, 54]. Each E3 ligase promotes colorectal carcinogenesis by targeting different substrates. Hence, it will be interesting to study whether there is a correlation between RING‐type E3 ligases and colon cancer. In a previous study, Peli3 did not show any oncogenic activity in lung cancer cells when compared to Peli1 [55]. This discrepancy may be attributed to the model system (mouse and human cell line) or target cell (macrophage and epithelial cells). Further studies are required to understand the exact role of different Peli proteins in the development of various cancers.

As E3 ubiquitin ligases are involved in the progression of various cancers, a valuable approach is to develop small molecules targeting ubiquitin ligases. Interestingly, E3 ligases have a high affinity to a specific substrate, unlike E1 or E2 subunits. Therefore, there are several approaches to identify specific inhibitors blocking the interaction of E3 ligase and its substrate or reducing ligase activity [56, 57]. For example, Nutlin‐3a and its derivatives induced cell cycle arrest and apoptosis by inhibiting MDM2‐mediated p53 degradation [57]. Another oncogenic Cdc20 subunit in the APC/C complex may be a therapeutic target for cancer treatment. Many compounds have been identified as potential anti‐cancer drugs by targeting Cdc20 [58, 59, 60]. Currently, there is no specific inhibitor to suppress the E3 ubiquitin ligase activity of Peli3. However, specific inhibitors for another Peli protein (Peli1) was found with E‐cadherin luciferase reporter screening using a natural compound library [61]. Through this screening, resistomycin was identified as a specific Peli1 inhibitor to suppress the development and metastasis of breast cancer by blocking the interaction of Peli1 with SNAIL/SLUG. Another study also demonstrated that Smadcin‐6, a Smad6‐derived peptide, interacts with Peli1 to disrupt Peli1‐mediated TLR4 signalling, and that it also showed therapeutic effects in lethal inflammatory disease and congenital Zika syndrome [62, 63]. In this study, Smadcin‐6 can also bind to Peli3 but not to Peli2 [62]. This suggests that Smaducin‐6 can be applied to a CAC animal model to determine whether this short peptide is effective in suppressing Peli3‐mediated inflammation and carcinogenesis. A novel protein degradation technique, PROTAC (Proteolysis targeting chimera), may potentially be applied in cancer therapy. Interestingly, several PROTACs have been developed based on specific interactions and degradation of E3 ubiquitin ligases [64, 65, 66]. By developing a specific Peli3‐interacting moiety, PROTAC can be used as a therapeutic strategy to suppress Peli3‐mediated CAC.

IRF4 is a transcription factor in inflammatory signalling and plays an essential role as a tumour promoter or suppressor depending on the cancer type or cell [67, 68, 69, 70]. IRF4 overexpression decreases Tregs stability, thereby increasing macrophage‐like transformation of Tregs, repressing the development of colorectal cancer cells, and exacerbating anti‐tumour immunosuppression [71]. As a tumour suppressor, RNF2 E3 ligase promotes proliferation of colon cancer cells by increasing IRF4 degradation through K48‐linked ubiquitination [72]. Additionally, IRF4 acts as a negative regulator of TLR4 signalling by modulating cIAP1/2 ubiquitination and expression of inflammatory cytokines in TNBS‐induced colitis [73]. Furthermore, MDP‐induced IRF4 inhibits polyubiquitination of TRAF6 and RICK, thereby reducing NF‐κB expression in a TNBS‐induced colitis model. These results suggest that colonic IRF4 expression induced by MDP administration can prevent and treat colitis [31]. Interestingly, our results showed that IRF4 mRNA expression was unchanged, but the protein level was decreased by ubiquitination‐dependent degradation of IRF4 in CAC (Figs 5 and 6). However, the importance of IRF4 levels in cancer progression remains unknown. Thus, identifying inhibitory chemicals or therapeutic techniques to increase or stabilise the level of IRF4 protein is important to suppress CAC development.

## 5 Conclusions

In this study, we demonstrated a novel pathological role of Peli3 in the development of CAC using Peli3‐deficient mice. We observed that Peli3 promotes colorectal tumorigenesis, with increased tumour burden and inflammatory signalling pathways. Mechanistic studies indicate that Peli3 enhances the TLR4‐mediated inflammation through degradation of IRF4, a negative regulator of TLR4. Our study suggests an important molecular link between Peli3 and colonic inflammation and carcinogenesis, and that Peli3 can be a therapeutic target in the prevention and treatment of CAC.

## Conflict of interest

The authors declare no conflict of interest.

## Author contributions

Y‐MK, H‐YK, HTHT and JK designed and performed the experiments. YJL generated the animal model. Y‐MK, H‐YK, HTHT and SH analysed the data and wrote the manuscript. SJK and SH proposed and supervised the project. All authors approved the final version of manuscript including the authorship list.

### Peer review

The peer review history for this article is available at https://www.webofscience.com/api/gateway/wos/peer-review/10.1002/1878-0261.13475.

## Supporting information


**Fig. S1.** Generation of Peli3 knockout (KO) mouse and protocol for azoxymethane (AOM)/dextran sulphate sodium (DSS)‐induced colitis‐associated colorectal cancer (CAC) model.
**Fig. S2.** Peli3 induces formation of aberrant crypt foci (ACF) at early stages of colitis‐associated colorectal cancer (CAC) progression.
**Fig. S3.** Infiltration of immune cells is reduced in Peli3‐depleted colitis tissues.
**Fig. S4.** Peli3 is required for inflammatory cytokine expression and dysbiosis during colitis‐associated colorectal cancer (CAC) development.
**Fig. S5.** Expression of IRF4 is unchanged by inflammatory stimuli.
**Fig. S6.** Absence of Peli3 protects against degradation of IRF4.
**Fig. S7.** Generation of IRF4‐knockdown peritoneal macrophages in Peli3 knockout cell.

**Table S1.** List of used PCR primer sequences for genotyping.
**Table S2.** List of used shRNA sequences in this study.
**Table S3.** List of used RT‐PCR primer sequences in this study.

## Data Availability

No datasets were generated or submitted related to this paper that is available in a public database.
