# Supplementary material for: Pellino 3 promotes the colitis‐associated colorectal cancer through suppression of IRF4‐mediated negative regulation of TLR4 signalling
Source: Mol Oncol. 2023 Jun 27;17(11):2380–95. doi: 10.1002/1878-0261.13475 (PMC10620127; doi:10.1002/1878-0261.13475)
Supplement: Supplementary file 1 — Fig. S1. Generation of Peli3 knockout (KO) mouse and protocol for azoxymethane (AOM)/dextran sulphate sodium (DSS)‐induced colitis‐associated colorectal cancer (CAC) model. Fig. S2. Peli3 induces formation of aberrant crypt foci (ACF) at early stages of colitis‐associated colorectal cancer (CAC) progression. Fig. S3. Infiltration of immune cells is reduced in Peli3‐depleted colitis tissues. Fig. S4. Peli3 is required for inflammatory cytokine expression and dysbiosis during colitis‐associated colorectal cancer (CAC) development. Fig. S5. Expression of IRF4 is unchanged by inflammatory stimuli. Fig. S6. Absence of Peli3 protects against degradation of IRF4. Fig. S7. Generation of IRF4‐knockdown peritoneal macrophages in Peli3 knockout cell. [file MOL2-17-2380-s001.pptx]

## Slide 1
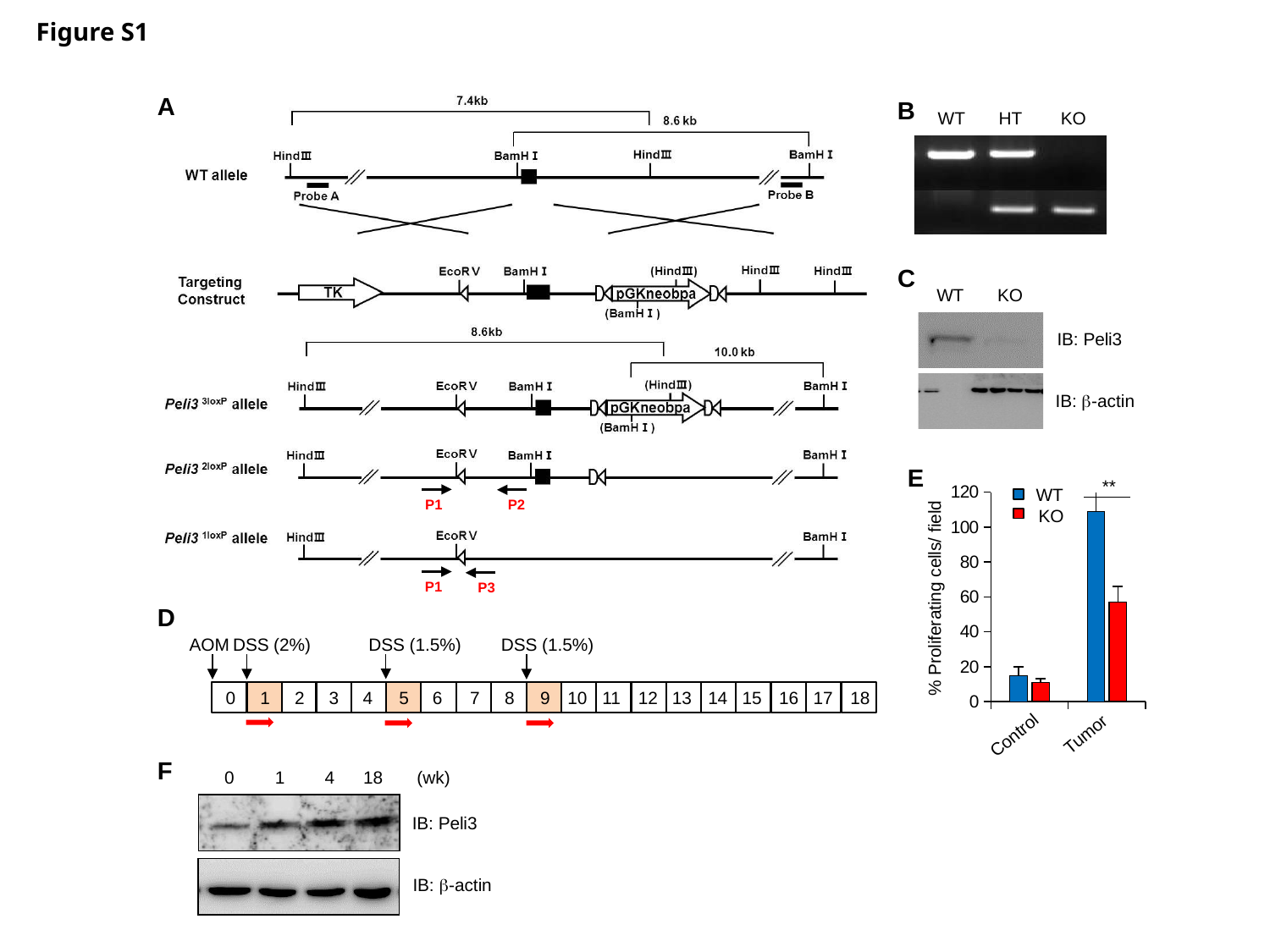

Figure S1
A
B
WT HT KO
C
WT KO
IB: Peli3
IB: b-actin
### Chart
| Category | WT | Peli3-/- |
|---|---|---|
| con | 15.0 | 11.0 |
| Tumor | 109.0 | 57.0 |E
**
WT
KO
P1
P2
P1
P3
% Proliferating cells/ field
D
AOM
DSS (2%)
DSS (1.5%)
DSS (1.5%)
0
2
3
4
6
7
8
10
11
12
13
14
15
16
17
18
1
5
9
Tumor
Control
F
0
1
4
18
(wk)
IB: Peli3
IB: b-actin

## Slide 2
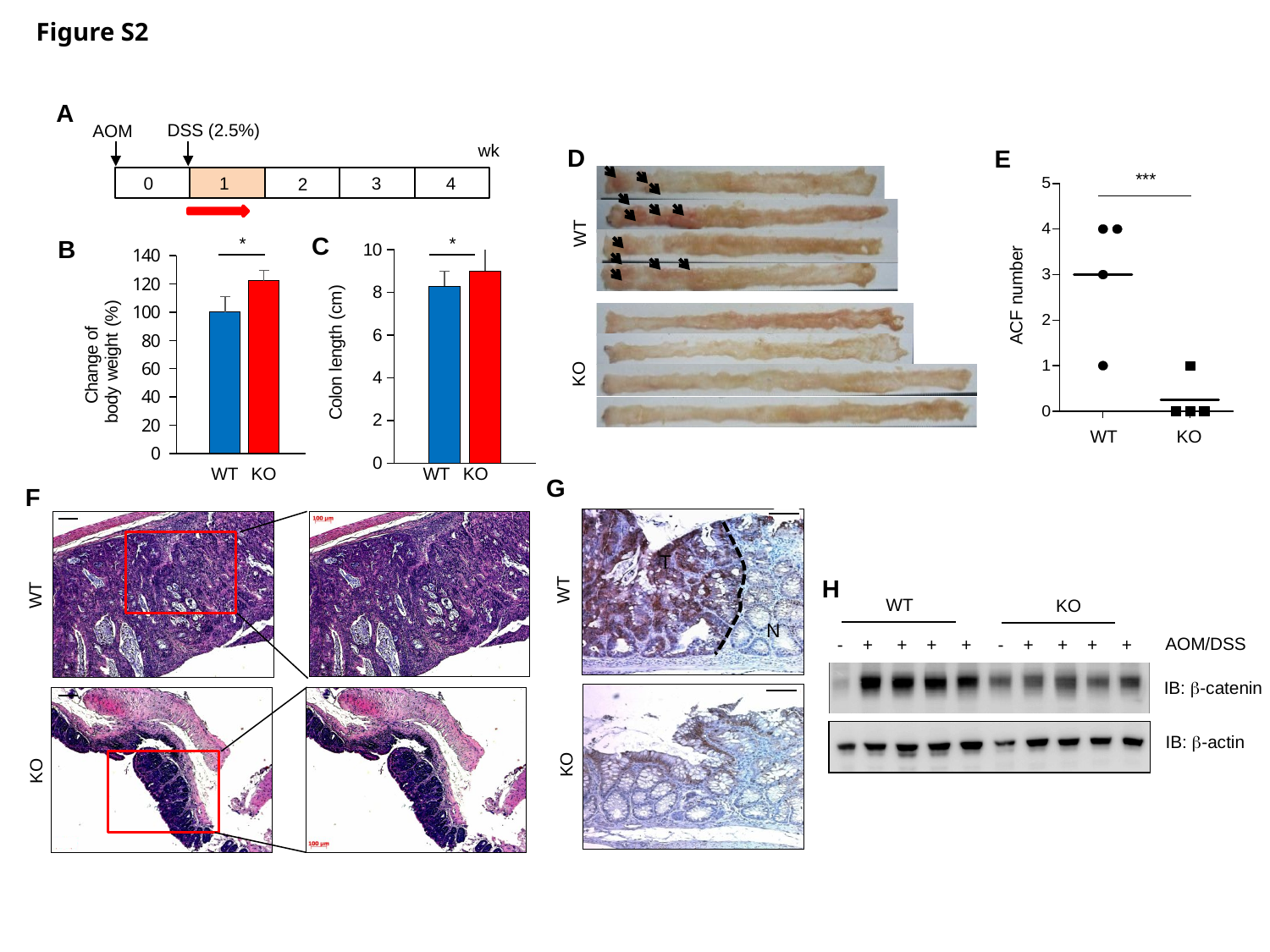

Figure S2
A
DSS (2.5%)
AOM
wk
D
E
***
0
1
3
4
2
WT
C
*
*
B
### Chart
| Category | | |
|---|---|---|
### Chart
| Category | | |
|---|---|---|ACF number
KO
WT
KO
WT
KO
WT
KO
G
F
T
H
WT
WT
WT
KO
N
AOM/DSS
 - + + + +
 - + + + +
IB: β-catenin
IB: β-actin
KO
KO

## Slide 3
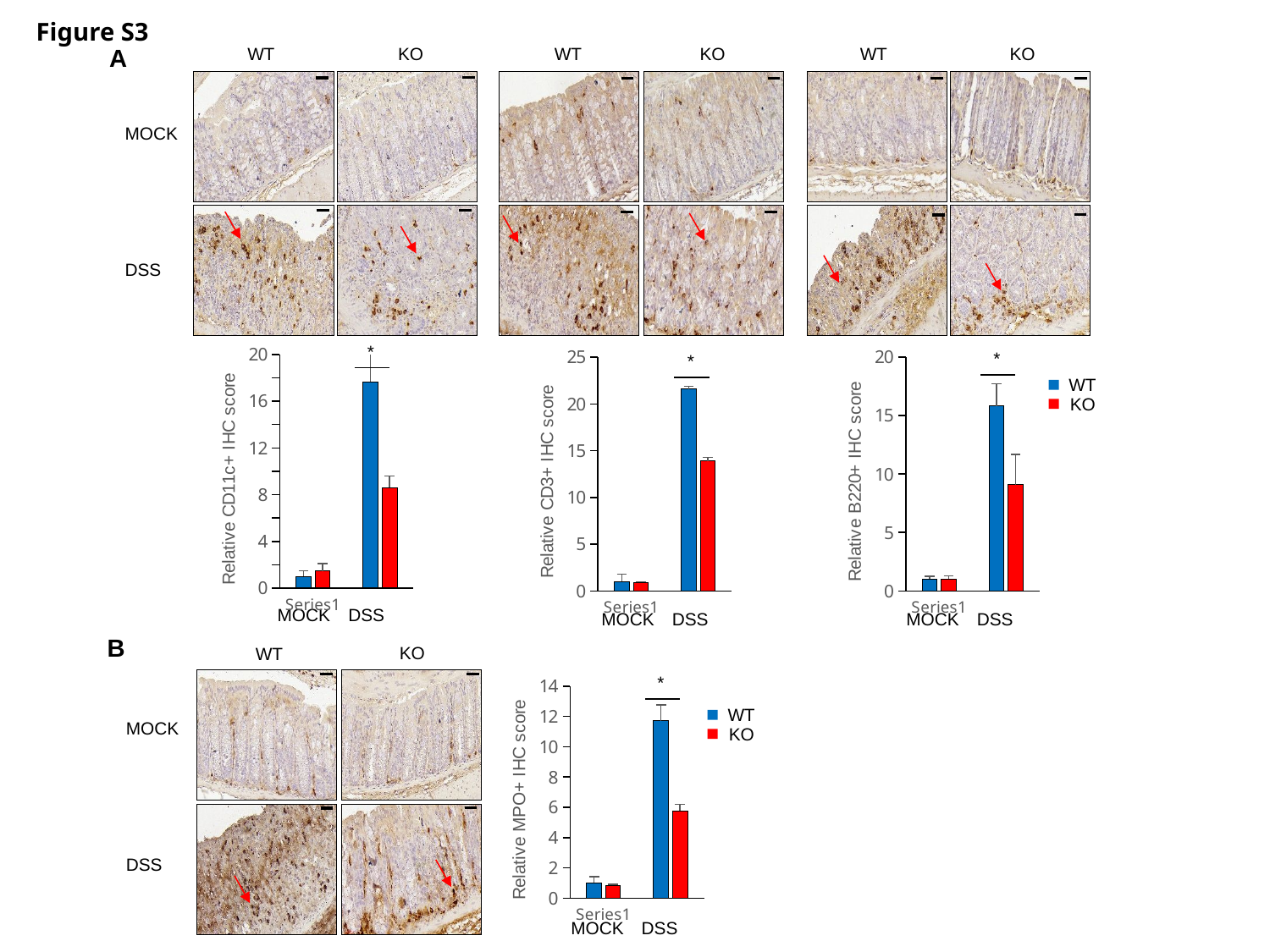

Figure S3
A
WT
KO
WT
KO
WT
KO
MOCK
DSS
*
### Chart
| Category | WT | KO |
|---|---|---|
| | 1.0 | 1.4506024096385541 |
| | 17.67228915662651 | 8.590361445783131 |
### Chart
| Category | WT | KO |
|---|---|---|
| | 1.0 | 0.9005524861878454 |
| | 21.62154696132597 | 13.91436464088398 |
### Chart
| Category | WT | KO |
|---|---|---|
| | 1.0 | 1.0265654648956357 |
| | 15.838709677419358 | 9.100569259962048 |*
*
WT
KO
MOCK
DSS
MOCK
DSS
MOCK
DSS
B
KO
WT
*
### Chart
| Category | WT | KO |
|---|---|---|
| | 1.0 | 0.8286713286713286 |
| | 11.702797202797203 | 5.745920745920746 |
WT
KO
MOCK
DSS
MOCK
DSS

## Slide 4
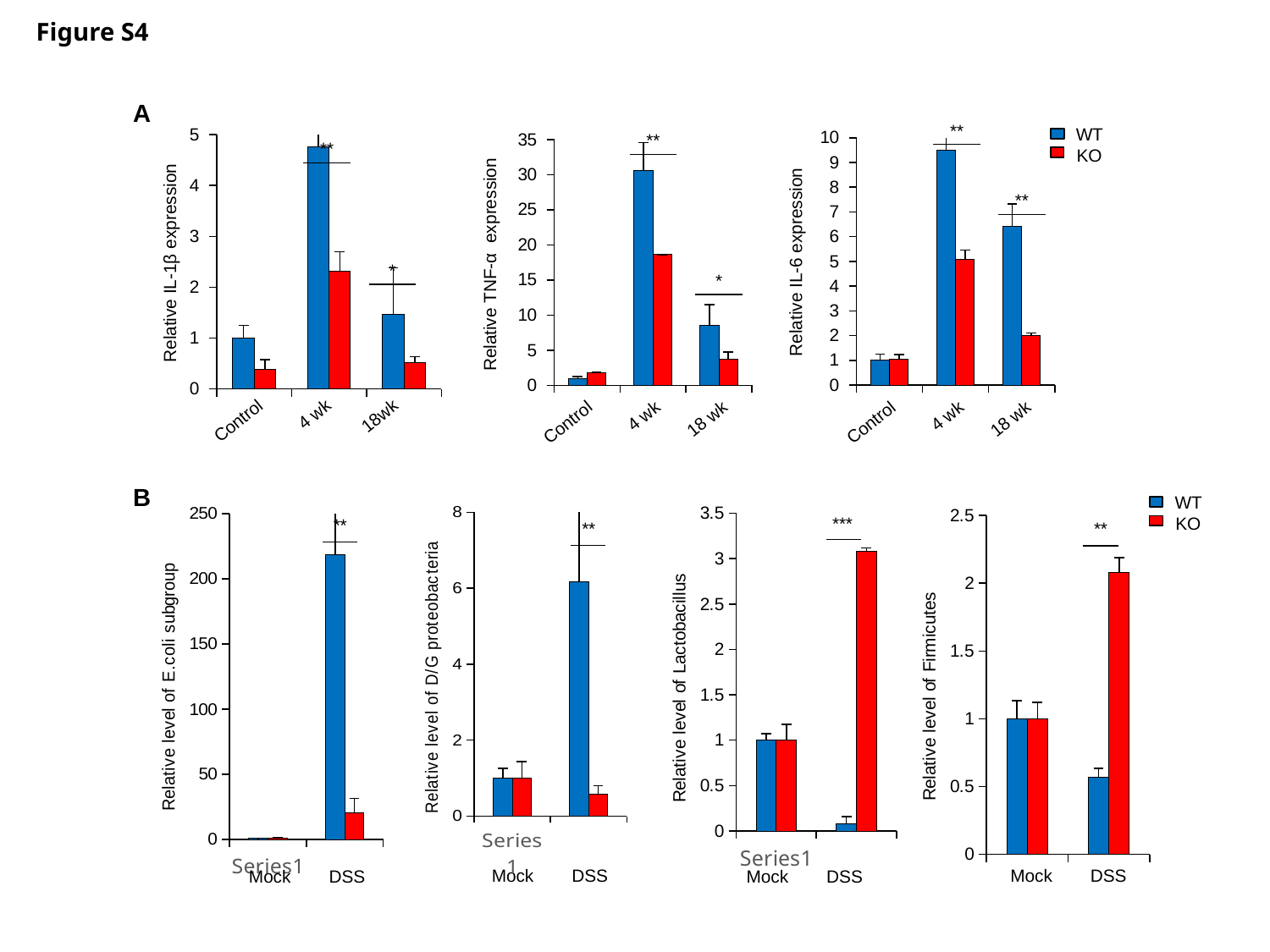

Figure S4
A
**
### Chart
| Category | WT | KO |
|---|---|---|
| control | 1.00170895573969 | 1.04255493098482 |
| 4W | 9.49925024011366 | 5.07925027263573 |
| 13W | 6.40708475997988 | 1.9931337161335 |WT
KO
### Chart
| Category | TNF-a | |
|---|---|---|
| control | 0.996782797979743 | 1.82494857824682 |
| 4W | 30.5670953215643 | 18.6083803353303 |
| 13W | 8.5903821677409 | 3.65898297521425 |
### Chart
| Category | WT | KO |
|---|---|---|
| Control | 0.999999464567513 | 0.388064874869547 |
| 4W | 4.7533784316087 | 2.30745687011656 |
| 13W | 1.46007920498761 | 0.524440895004568 |**
**
**
Relative IL-1β expression
 Relative IL-6 expression
 Relative TNF-α expression
*
*
4 wk
4 wk
4 wk
18wk
18 wk
18 wk
Control
Control
Control
B
WT
KO
### Chart
| Category | | |
|---|---|---|
| | 1.0 | 1.0 |
| | 218.62479384764484 | 20.43231339958436 |
### Chart
| Category | WT | KO |
|---|---|---|
| | 1.0 | 1.0 |
| | 6.176710343920801 | 0.5864415815922581 |
### Chart
| Category | WT | KO |
|---|---|---|
| | 1.0 | 1.0 |
| | 0.08021671856901219 | 3.08453072395847 |
### Chart
| Category | WT | KO |
|---|---|---|
| MOCK | 1.0 | 1.0 |
| DSS | 0.566347 | 2.07633469520944 |***
**
**
**
DSS
DSS
Mock
Mock
DSS
Mock
DSS
Mock

## Slide 5
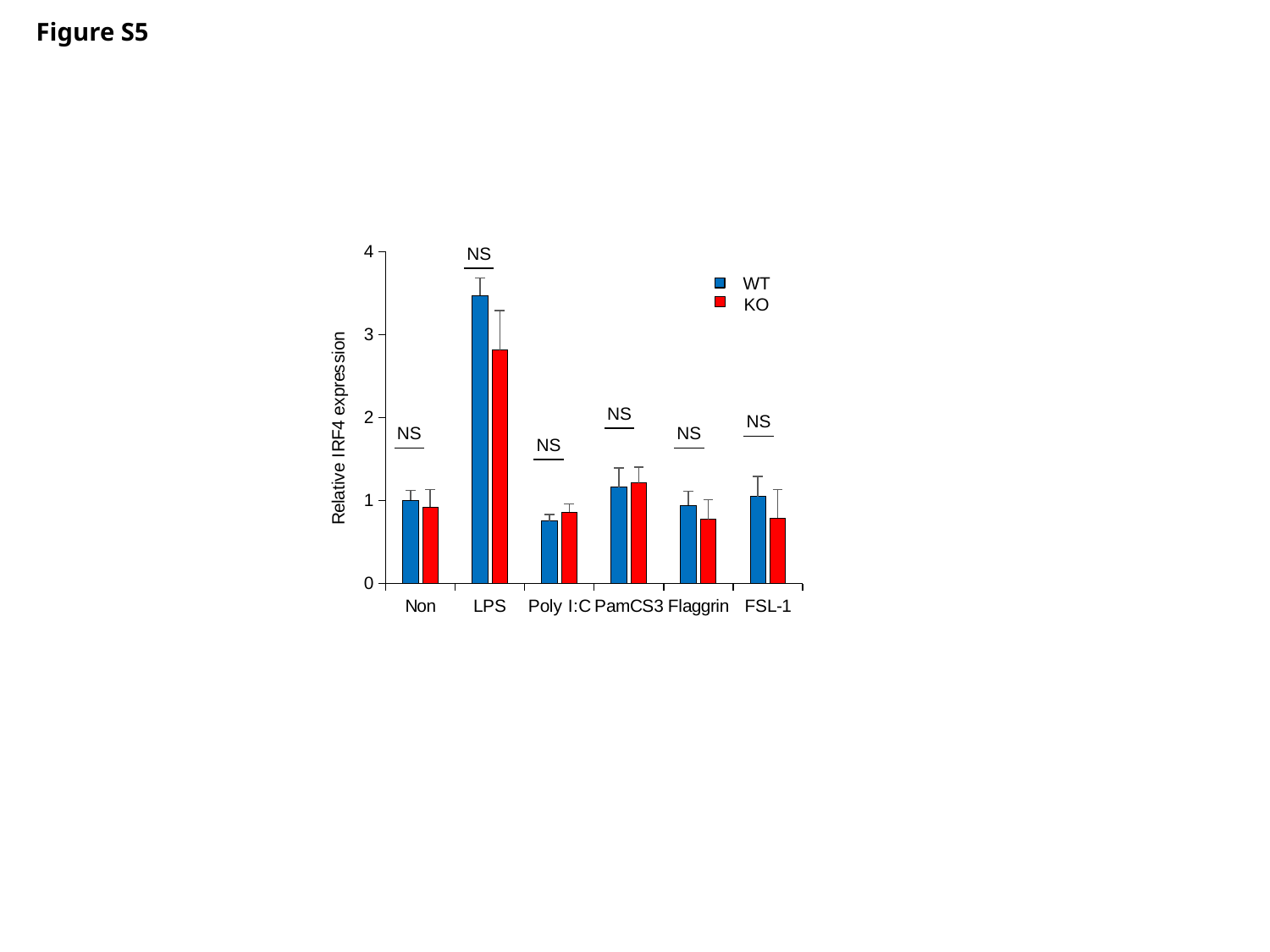

Figure S5
### Chart
| Category | WT | KO |
|---|---|---|
| Non | 1.0 | 0.92 |
| LPS | 3.47 | 2.81 |
| Poly I:C | 0.75 | 0.86 |
| PamCS3 | 1.16 | 1.21 |
| Flaggrin | 0.94 | 0.77 |
| FSL-1 | 1.05 | 0.79 |NS
WT
KO
NS
NS
NS
NS
NS

## Slide 6
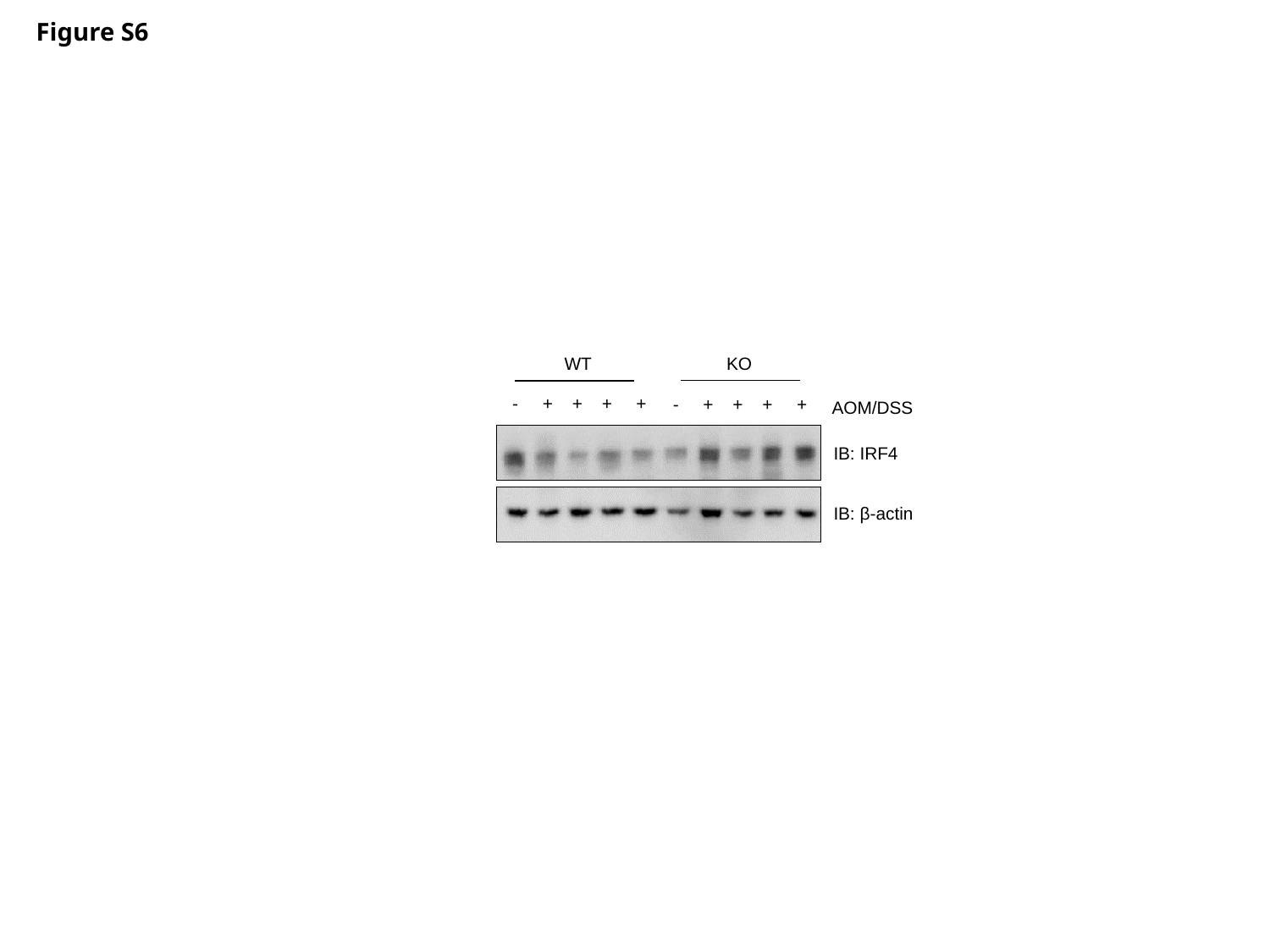

Figure S6
WT
KO
 - + + + +
 - + + + +
AOM/DSS
IB: IRF4
IB: β-actin

## Slide 7
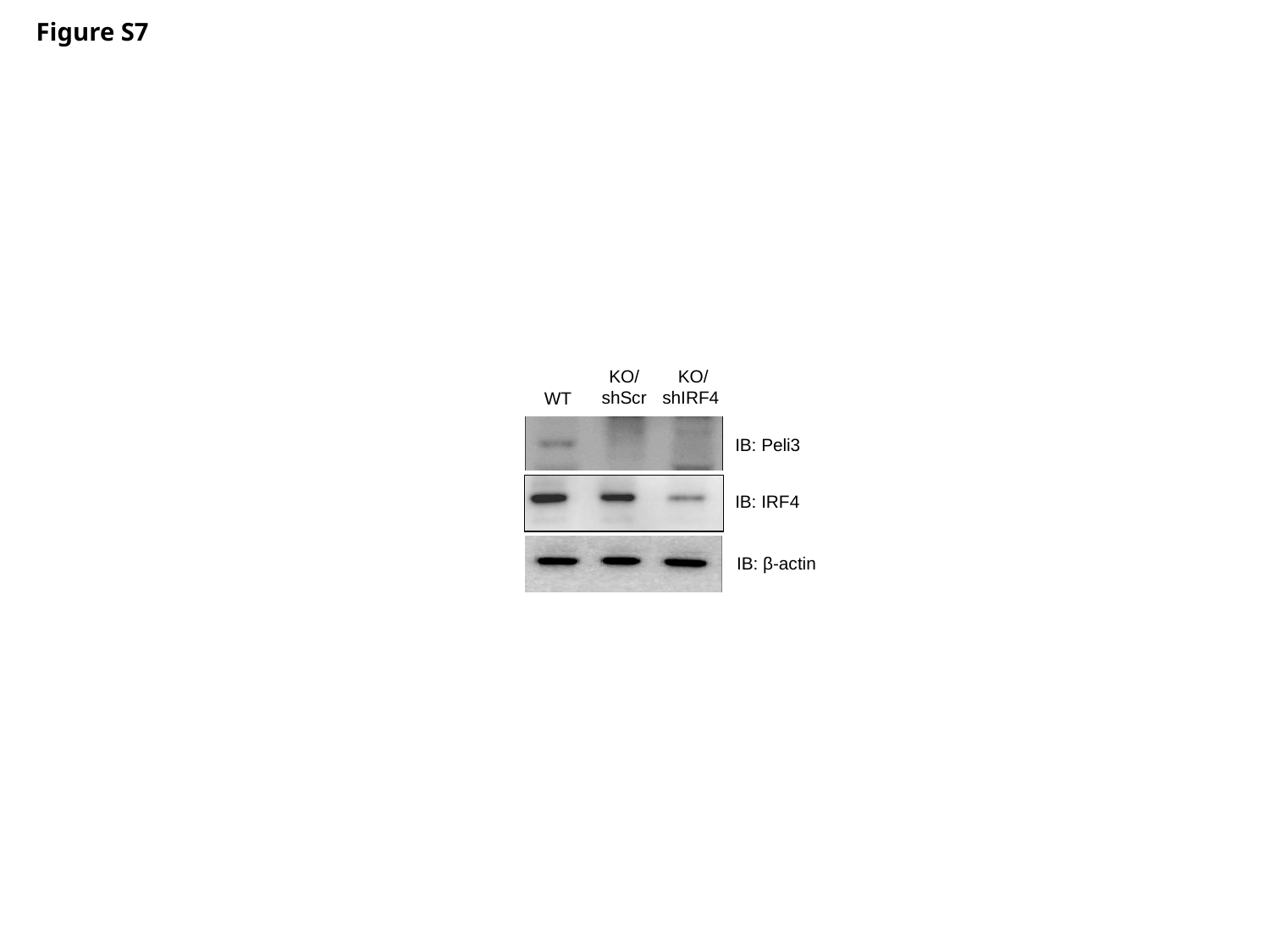

Figure S7
KO/
shScr
KO/
shIRF4
WT
IB: Peli3
IB: IRF4
IB: β-actin
