# Supplementary material for: Pellino 3 promotes the colitis‐associated colorectal cancer through suppression of IRF4‐mediated negative regulation of TLR4 signalling
Source: Mol Oncol. 2023 Jun 27;17(11):2380–95. doi: 10.1002/1878-0261.13475 (PMC10620127; doi:10.1002/1878-0261.13475)
Supplement: Supplementary file 2 — Table S1. List of used PCR primer sequences for genotyping. Table S2. List of used shRNA sequences in this study. Table S3. List of used RT‐PCR primer sequences in this study. [file MOL2-17-2380-s002.docx]

**Supplementary figure legends**

**Fig. S1.** Generation of Peli3 knockout (KO) mouse and protocol for azoxymethane (AOM)/dextran sulfate sodium (DSS)-induced colitis-associated colorectal cancer (CAC) model. (A) Peli3 whole KO mice were generated by mating Peli3^2loxP^ mice with β-actin Cre mice. (B and C) The expression of Peli3 mRNA or protein was measured with RT-PCR or Western blot in Peli3 wild type or KO mice. (D) Protocol for AOM/DSS-induced CAC model. Mice were injected with 8 mg/kg AOM once, and DSS was administered for three cycles every 5 days. (E) PCNA-positive cells were counted from several fields of each slide and presented with the % of proliferating cells/field. Related with Fig. 1F. ***p* < 0.01. (F) To measure the expression of Peli3 protein during CAC development, colon tissues were harvested after different times of AOM/DSS treatment and analyzed with Peli3 antibody by western blotting.

**Fig. S2.** Peli3 induces formation of aberrant crypt foci (ACF) at early stages of colitis-associated colorectal cancer (CAC) progression. (A) Experimental protocol for formation of ACF. Mice were injected with AOM once and administered with 2.5% dextran sulfate sodium for 5 days (n=6 per group). Mice were sacrificed after 4 weeks, and colonic tissues were collected for further analysis. (B and C) Body weight and colon length of Peli3 wild type (WT) or knockout (KO) mice were measured. (D) Representative images of the colon from the cecum to the proximal rectum of Peli3 WT or KO mice. Arrowheads indicate AFC. (E) The number of ACF was counted and compared between Peli3 WT and KO mice. (F) Representative images for hematoxylin–eosin staining of colonic tissues are shown. (G) Proliferating cells were evaluated with PCNA staining of the normal or tumor regions in each mouse. N, normal tissue. (H) Expression of β-catenin was measured with lysates of colonic tissue. The band intensity was compared with the one of β-actin. Scale bar, 100 μm. Results are presented as a mean ± standard deviation from three independent experiments. **p* < 0.05, ****p* < 0.001.

**Fig. S3.** Infiltration of immune cells is reduced in Peli3-depleted colitis tissues. (A) Representative immunohistochemical images of immune cells were shown (n=3 per group). Slides were stained with anti-CD11c for dendritic cells, anti-CD3 for T cells or anti-B220 for B cells. The relative level of infiltrated immune cells was normalized with untreated WT image. (B) The expression level of myeloperoxidase (MPO) was detected with immunohistochemical staining in control or DSS-treated colon tissues. The relative level of MPO^+^ cells was normalized with untreated WT samples. Scale bar, 30 μm. **p* < 0.05.

**Fig. S4.** Peli3 is required for inflammatory cytokine expression and dysbiosis during colitis-associated colorectal cancer (CAC) development. (A) The expression level of inflammatory cytokine genes in colonic tissues of Peli3 wild type or knockout mice was detected using qRT–PCR at 4 or 18 weeks of AOM/DSS treatment (n=3 per group). Levels of cyclophilin gene were used as normalization control. (B) Change of microbiome distribution was detected with cecum DNA using qRT-PCR after DSS treatment (n=3 per group). Relative level of each microbiome group was normalized with level of eubacteria. **p* < 0.05; ***p* < 0.01; ***<0.001.

**Fig. S5.** Expression of IRF4 is unchanged by inflammatory stimuli. The expression level of IRF4 gene was detected using qRT–PCR after treatment with various toll-like receptor ligands in peritoneal macrophages. Levels of cyclophilin gene were used as normalization control.

**Fig. S6.** Absence of Peli3 protects against degradation of IRF4. To measure the level of IRF4 in colonic tissues, proteins were extracted after treatment with AOM/DSS for 4 weeks. Then, whole protein was extracted after grinding colonic tissues, and levels of IRF4 were detected with the specific antibody.

**Fig. S7.** Generation of IRF4-knockdown peritoneal macrophages in Peli3 knockout cell. Knockdown cells were generated with lentiviral transduction of shRNA against IRF4 into Peli3 knockout cells. Then, expression levels of each protein were analyzed with Western blotting. The band intensity of each protein was normalized with that of β-actin.

**Table S1. List of used PCR primer sequences for genotyping**

| Primer name | PCR primer sequences |
| --- | --- |
| gPeli3-1 | TCA CCA GTC TCC CTC CTT GGT ATC |
| gPeli3-2 | GAC AAC TTG CTG AAG TAG AAT TGG |
| gPeli3-3 | CAT TCT GAG CAC AGT GGG AGA AGA |

**Table S2. List of used shRNA sequences in this study**

| Primer name | shRNA sequences |
| --- | --- |
| shIRF4-F | CCG GCT AGC CAG ACA ACT GTA TTA CCT CGA GGT AAT ACA GTT GTC TGG CTA GTT TTT G |
| shIRF4-R | AAT TCA AAA ACT AGC CAG ACA ACT GTA TTA CCT CGA GGT AAT ACA GTT GTC TGG CTA G |

**Table S3. List of used RT-PCR primer sequences in this study**

| Primer name | RT-PCR primer sequences |
| --- | --- |
| mTNF-F | CAT GAC ATC TGG CTT CAC TC |
| mTNF-R | TGG GAG TAG ACA AGG TAC AAC CC |
| mIL6-F | GAG GAT ACC ACT CCC AAC AGA CC |
| mIL6-R | AAG TGC ATC ATC GTT GTT CAT ACA |
| mIL1b-F | AGG CAG GCA GTA TCA CTC ATT GT |
| mIL1b-R | GGA AGG TCC ACG GGA AAG AC |
| mPeli3-F | CTC ATC GTT CTG GGC TAC |
| mPeli3-R | CATGACATCTGGCTTCACTC |
| mIRF4-F | GCC CAA CAA GCT AGA AAG |
| mIRF4-R | TCT CTG AGG GTC TGG AAA CT |
| Eubacteria F | AAA CTC AAA KGA ATT GAC GG |
| Eubacteria R | CTC ACR RCA CGA GCT GAC |
| E. coli F | CAT GCC GCG TGT ATG AAG AA |
| E. coli R | CGG GTA ACG TCA ATG AGC AAA |
| Proteobacteria F | GCT AAC GCA TTA AGT RYC CCG |
| Proteobacteria R | GCC ATG CRG CAC CTG TCT |
| Lactobacillus F | CGA TGA GTG CTA GGT GTT GGA |
| Lactobacillus R | CAA GAT GTC AAG ACC TGG TAA G |
| Firmicutes F | GGA GYA TGT GGT TTA ATT CGA AGC A |
| Firmicutes R | AGC TGA CGA CAA CCA TGC AC |
